# Crystal structures of FNIP/FGxxFN motif-containing leucine-rich repeat proteins

**DOI:** 10.1038/s41598-022-20758-8

**Published:** 2022-09-30

**Authors:** Trevor Huyton, Mamta Jaiswal, Waltraud Taxer, Matthias Fischer, Dirk Görlich

**Affiliations:** 1 Department of Cellular Logistics, Max Planck Institute for Multidisciplinary Sciences, Göttingen, Germany; 2 Department of Biomolecular Mechanisms, Max Planck Institute for Medical Research, Heidelberg, Germany

**Keywords:** Biochemistry, Biological techniques, Biophysics, Computational biology and bioinformatics, Evolution, Structural biology

## Abstract

The *Cafeteria roenbergensis* virus (Crov), *Dictyostelium*, and other species encode a large family of leucine-rich repeat (LRR) proteins with FGxxFN motifs. We determined the structures of two of them and observed several unique structural features that set them aside from previously characterized LRR family members. Crov588 comprises 25 regular repeats with a LxxLxFGxxFNQxIxENVLPxx consensus, forming a unique closed circular repeat structure. Novel features include a repositioning of a conserved asparagine at the middle of the repeat, a double phenylalanine spine that generates an alternate core packing arrangement, and a histidine/tyrosine ladder on the concave surface. Crov539 is smaller, comprising 12 repeats of a similar LxxLxFGxxFNQPIExVxW/LPxx consensus and forming an unusual cap-swapped dimer structure. The phenylalanine spine of Crov539 is supplemented with a tryptophan spine, while a hydrophobic isoleucine-rich patch is found on the central concave surface. We present a detailed analysis of the structures of Crov588 and Crov539 and compare them to related repeat proteins and other LRR classes.

## Introduction

*Cafeteria roenbergensis* virus (Crov) is a giant virus in the family *Mimiviridae* that infects marine microplankton such as the bicosoecid flagellate *Cafeteria* sp.^1–3^*.* The virus was originally isolated during the 1980s from seawater samples taken in the Gulf of Mexico^3^. Since its isolation, the genomics and structure of the virus have been well described. Crov has an exceptionally large genome with 692 kilobase pairs of double-stranded DNA and > 500 predicted genes^1,2,4–8^. Its capsid has a diameter of 3000 Å and represents one of the largest structures reconstructed by cryo-electron microscopy^9^. Crov is one of several giant viruses that are parasitized by smaller DNA viruses called virophages^5^.

Our interest in Crov arose after discovering that it contains multiple proteins with long repetitive motifs^1,10^, which we will refer to as FGxxFN repeat proteins. Specifically, these motifs contain FG dipeptides that are best known from intrinsically disordered FG nucleoporin repeat domains^11,12^, which can cohesively interact with each other and condense into an FG phase^13^. This FG phase functions as a sieve-like permeability barrier of nuclear pore complexes (NPCs). It allows for controlled transport between the nuclear and cytoplasmic compartments via shuttling nuclear transport receptors that interact with FG motifs (for a detailed review, see^14^). We have also shown that isolated nucleoporin FG repeats form hydrogels that reproduce the permeability properties of NPCs remarkably well^13–17^. However, structural information on FG-FG interactions is extremely limited due to the technical problems of measuring transient hydrophobic interactions. We therefore reasoned that the structure of a soluble FG-containing protein might give some further insights into the interactions and stacking of phenylalanine residues within FG-containing peptides.

The FGxxFN repeat proteins belong to a larger family termed FNIP proteins (Pfam PF05725), which in turn appear related to the large and well-studied Leucine-rich repeat (LRR) family of proteins. LRRs are present in over 60 000 proteins identified in viruses, bacteria, archaea, and eukaryotes. The structures of many LRR proteins are known. The arrangement of tandem LRR repeats gives rise to a typical curved α/β-solenoid structure. Many LRR-containing proteins function as scaffolds that participate in protein–protein or protein–ligand interactions and help to regulate processes like signal transduction, cell adhesion, DNA repair, recombination, transcription, RNA processing, disease resistance, apoptosis, and innate immune response in mammals, fish, and plants^18^. LRR-containing proteins include RNAse inhibitors, toll-like receptors (TLRs), hormone receptors, tyrosine kinase receptors, cell-adhesion molecules, bacterial virulence factors, and many others^18,19^. LRRs vary in consensus sequence and repeat length, ranging typically between 20 and 30 residues.

LRRs are divided into a highly conserved and a variable segment. The conserved segment of the prototypic LRR proteins consists of an 11-residue LxxLxLxxNxL stretch or a 12-residue LxxLxLxxCxxL stretch, (where L = leucine/isoleucine/valine, N = asparagine/cysteine/threonine/serine, C = cysteine/ and x = any amino acid). Traditionally, LRRs are divided into eight classes. These include (1) typical, (2) ribonuclease inhibitor (RI)-like, (3) cysteine-containing (CC), (4) plant-specific (PS), (5) SDS22-like, (6) bacterial, (7) TpLRR (*Treponema pallidum* LRR), and (8) IRREKO^20–22^.

The FNIP family (Pfam PF05725) so far comprises > 2000 family members, including many repeat proteins from Crov^1,10^, Megavirus baoshan^23^, and the slime mold genera *Polysphondylium* and *Dictyostelium*^24,25^. The FNIP repeats were named after their 22 residues long hxxhxhxxx**FN**xxhxxx**IP**xx consensus (with h being hydrophobic residues). The here described FGxxFN repeats (including the Crov repeat proteins) are a major subfamily of them. However, to our knowledge, no experimental structures of FGxxFN or FNIP leucine-rich repeats have been elucidated so far. We now close this gap and report the crystal structures of Crov588 (with an LxxLxFGxxFNQxIxENVLPxx consensus) and Crov539 (with an LxxLxFGxxFNQPIExVxWPxx consensus) that are quite representative of the two subfamilies of Crov FGxxFN repeat proteins (supplementary sequence file 1). The structures reveal a so far unique hydrophobic packing with a buried phenylalanine spine and thus define a new sub-class of LRR proteins.

## Results

### Recombinant expression of Crov LRR proteins and their thermostability

**Figure 1:**
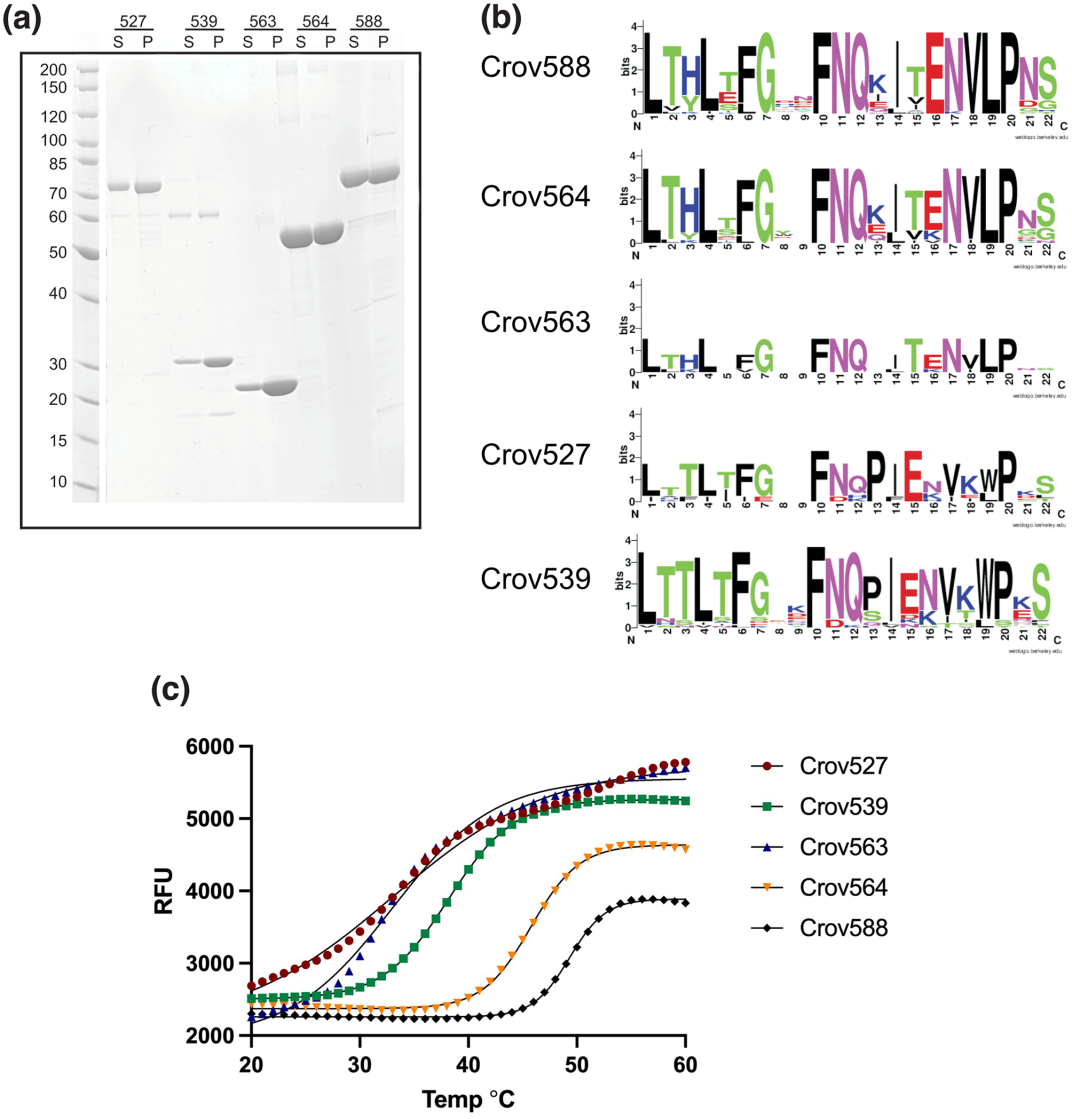
Properties of selected FGxxFN LRR proteins from Cafeteria roenbergensis virus. (**a**) SDS gel depicting soluble (S) and pellet (P) fractions in a heat stability assay, following heat denaturation at 97 °C for 5 min. (**b**) Logo plots of the corresponding 22-residue FGxxFN repeats. See supplementary sequence file for a comprehensive repeat annotation for all ~ 70 Crov FGxxNN repeat proteins. (**c**) Thermofluor measurements of unfolding. Calculated V50 melting temperature values are: Crov527 Tm 30.1 °C, Crov539 Tm 38.2 °C, Crov563 Tm 32.9 °C, Crov564 Tm 45.8 °C, Crov588 Tm 49.3 °C.

Initial analysis of the Crov genome identified ~ 30 genes encoding FGxxFN/ FNIP repeat-containing proteins^1^. Recent re-sequencing using long-read technology^10^ resolved additional repeat clusters and increased this number to ~ 70 (for repeat annotations, see Supplementary sequence file 1). Using codon-optimized gene constructs, we expressed and purified five of these proteins in *E. coli*: Crov527, Crov539, Crov563, Crov564, and Crov588 (Fig. 1a). An alignment of the repeat sequences for all five overexpressed Crov repeat proteins and their corresponding Logo plots (https://weblogo.berkeley.edu/logo.cgi) for their regular repeats (22 residues) within these sequences is presented in Fig. 1b.

Initial heat denaturation experiments at 97 °C for 5 min using purified protein indicated high thermal stability for all the Crov FGxxFN LRR proteins with ~ 25% of Crov527, 539, and 563 remaining in the supernatant after centrifugation. Crov564 and Crov588 remained soluble to even ~ 50% after heat treatment. Thermofluor assays, using SYPRO orange as an unfolding indicator, revealed, however, lower melting temperatures of 30–48 °C (Fig. 1c). We interpret this as efficient renaturation following melting, with refolding of Crov564 and 588 being more efficient than that of Crov527, 539, or 563. The relatively low initial Tm values for Crov539 and 563 were however surprising despite the standard growth temperature of 20–25 °C for *Cafeteria* sp. Many repeat proteins contain capping motifs which serve to shield the hydrophobic core from solvent and maintain structural integrity^26^. Other chemical denaturation studies on the LRR protein pp32 have shown dependence on the capping motifs^27,28^. The capping motifs from the selected Crov FGxxFN LRR proteins show little homology and this could be a reason for their distinct differences in their thermal stability (Fig. 1c). This becomes even more apparent when looking at the full Crov FGxxFN repeat proteins list (supplementary sequence file 1). The capping regions often (but not always) contain duplicate FN motifs features. The repeat patterns also show some variability, with Crov588 and Crov539 defining two sub-classes, differing foremost in the C-terminal half of the repeat.

### Structure determination of Crov588

We were able to obtain well-diffracting crystals of the purified Crov588 protein. We determined its structure using a combination of W-SAD phasing on crystals soaked with an Anderson-Evans polyoxotungstate [TeW_6_O_24_]^6−^ (TEW) cluster (Supplementary Figure 1), which is known as an effective phasing tool^29^. Soaking with tungstate appeared to induce a change in spacegroup (P12_1_1 > P2_1_2_1_2_1_) and reduced diffraction to 3.0 Å resolution. Initially, the tungstate structure was solved in spacegroup P2_1_2_1_2_1_. However, it became clear at later stages of building and refinement that P12_1_1 is the true spacegroup and that the spacegroup ambiguity was caused by a disorder in the termini due to the binding of clusters at the N/C-terminal interface of the circular structure. The structure of the native data was subsequently solved by molecular replacement using a partial model, and capping motifs at the termini were rebuilt (see methods; Table 1).

**Table 1:** Data collection and refinement statistics.

Structure PDB code	Crov588 Native (6NYR)	Crov588 Tungstate (6NYS)	Crov539 Native (7ZAL)
**Data collection**			
Wavelength (Å)	1.0	1.2108	1.0
Space group	P12_1_1	P12_1_1	P22_1_2_1_
Cell dimensions			
*a*, *b*, *c* (Å)	65.4, 172.6, 92.3	65.2, 171.7, 92.0	101.0, 113.4, 236.2
α, β, γ (°)	90, 94.7, 90	90, 90.1, 90	90, 90, 90
Resolution (Å)	49.09–2.43 (2.48–2.43)	46.02–3.1 (3.31–3.1)	49.46–2.73 (2.79–2.73)
R*merge*	0.1253 (1.14)	0.2419 (1.79)	0.123 (2.85)
R*p.i.m*	0.0525 (0.48)	0.067 (0.49)	0.034 (0.78)
CC_1/2_	0.99 (0.67)	0.98 (0.61)	0.99 (0.44)
Mean* I*/σ*I*	11.59 (1.44)	15.11 (1.15)	14.4 (1.0)
Completeness (%)	99.56 (99.95)	99.8 (99.92)	99.7 (95.0
Multiplicity	6.8 (6.5)	13.8 (13.6)	13.8 (13.6)
**Refinement**			
Resolution (Å)	49.09–2.43	46.02–3.1	49.44–2.73
No. of reflections	76,129 (7384)	36,693 (3654)	68,959 (4858)
*R*_work_ (%)	0.2204 (0.306)	0.2400 (0.351)	0.2197 (0.360)
*R*_free_ (%)	0.2568 (0.306)	0.2975 (0.415)	0.2666 (0.377)
No. of atoms			
Protein	11,110	11,086	14,496
Ligand/ion	30	465	0
Water	814	0	304
Mean* B* values (Å^2^)	56.84	116.5	46.59
Protein	57.29	109.49	45.87
Ligand/ion	75.95	284.11	N/A
Water	49.99	N/A	80.85
R.m.s. deviations			
Bond lengths (Å)	0.004	0.005	0.005
Bond angles (°)	1.04	1.03	1.11
Ramachandran analysis (%)			
Preferred	90.56	88.7	94.66
Allowed	9.22	10.78	5.34
Outliers	0.22	0.52	0.00

The refined structure from native data at 2.4 Å resolution contains two molecules in the asymmetric unit (see Table 1). The two copies superpose poorly with a root-mean-square deviation (RMSD) of 2.06 Å between the equivalent Cα positions of residues 2–680. This is predominantly due to a more twisted reorientation of the terminal capping regions within molecule B. Chain A from the native structure shows the best order. It was therefore chosen as the reference molecule for detailed structural analyses.

### Overall structure of Crov588, a novel LRR fold

**Figure 2:**
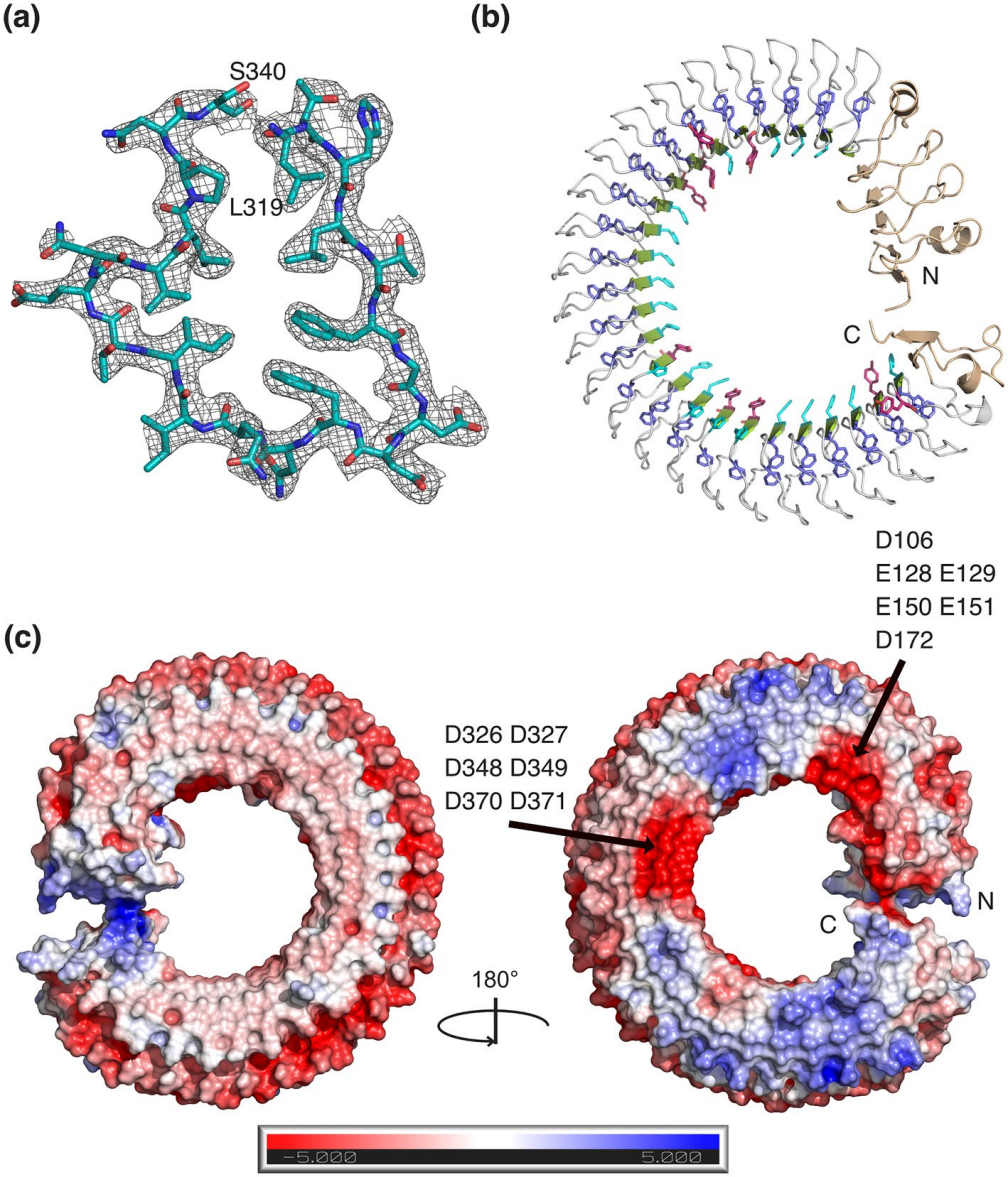
Structure of the Crov588 protein. (**a**) A zoomed view of an individual repeat is presented with 2Fo-Fc density contoured at 1.0σ. (**b**) Ribbon representation of the Crov588 structure. Highlighted are N and C-terminal capping repeats (wheat), the double phenylalanine spine (slate) histidine repeats (cyan) and tyrosine repeats (magenta). (**c**) Charged surface features of the Crov588 structure calculated with APBS (Pymol), highlighting the pronounced negatively charged patches in the N-terminal and middle of the structure.

The Crov588 structure reveals a compact core of 25 regular, 22 residues long LRRs flanked by N- and C-terminal capping modules that build a circular structure. At the N-terminus, four irregular 21–24 residue repeat sequences cap the structure, while a flanking strand extends to contact the C-terminus. The C-terminus is capped by a single 24 residue repeat and flanking strand. To our knowledge, this represents a unique LRR structure as it is the first “fully closed/circular” structure of a native LRR protein, where the N and C-terminal caps interact (Fig. 2). The circular LRR structure is ~ 90 Å in diameter with a central hole of ~ 45 Å.

### Sequence conservation within the Crov588 FGxxFN repeats

The 25 core LRRs are, in comparison to other LRR proteins, almost identical throughout the whole protein and contain a very highly conserved sequence motif LxxLxFGxxFNQxIxENVLPxx (where x is a variable residue) and this appears representative of the first sub-class of Crov FGxxFN repeat proteins (supplementary sequence file 1).

### Structural homology between Crov588 and other LRR-containing proteins

In almost all LRR structures studied, the highly conserved segments (LxxLxLxx**N**xL or LxxLxLxx**C**xxL) form a short β‐strand, which is completely, or almost completely, conserved. This feature positions an asparagine ladder, forming a continuous network of hydrogen bonds between consensus asparagines and neighboring backbone oxygens^30^. In FGxxFN repeat proteins, the conservation of this motif has deteriorated to only the first six residues (**L**xx**L**x**F**GxxFNxxIxxxxLPxx) before accommodation of the restricted peptide sequence that allows the double phenylalanine spine. The two residues between the FG and FN motifs are the most variable in all repeat proteins from Crov, its virophage mavirus, and *Dictyostelium*^24^. The asparagine ladder and subsequent continuous network of hydrogen bonds between repeats are conserved but have been shifted from position 9 in the regular LRR to position 12 in FGxxFN repeats. This stacking arrangement appears highly optimized and likely reflects an important protein folding constraint. In the archetypical LRR structure of ribonuclease inhibitor (pdb 1DFJ), the convex region of the repeat forms an α-helix. In other structures, such as the InlB structure (pdb 1D0B), loops or 3_10_-helices combined with highly ordered water molecules form a complex hydrogen bonded spine along the convex face between the adjacent LRR repeats^31^. Similarly, in the Crov588 structure, tightly bound water molecules participate in hydrogen bonding and stabilize the irregular loop structure along the convex surface of the structure at residue position 14 between the repeats.

### An unusual double phenylalanine spine

**Figure 3:**
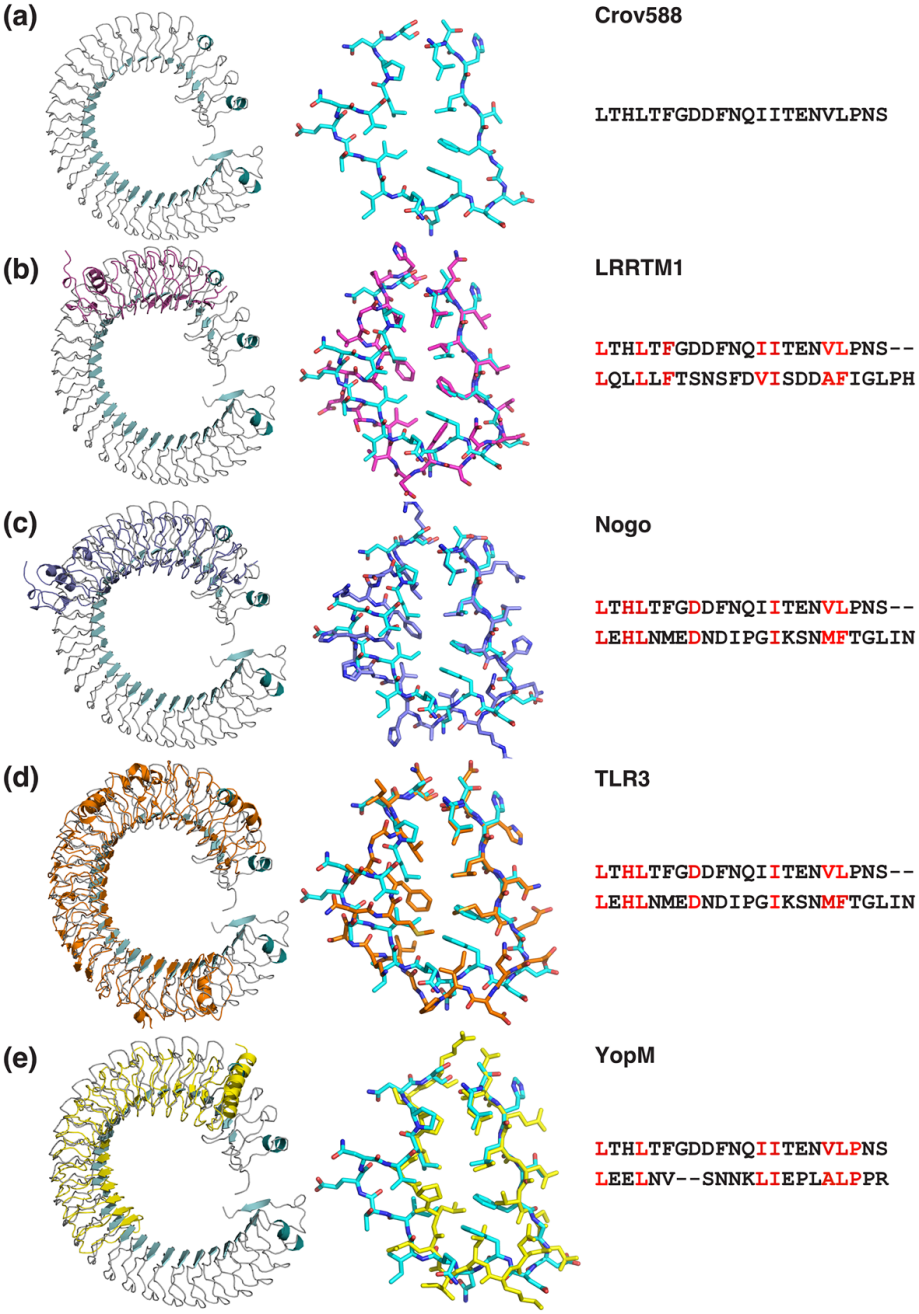
Overlay of Crov588 with structurally similar LRR-containing proteins. Side-by-side view of structures and aligned LRR motifs for (**a**) Crov588, (**b**) LRRTM1 (5Y30), (**c**) Nogo Receptor (1OZN), (**d**) TLR3 (1ZIW) and (**e**) YopM (1JL5).

A single spine formed by consecutive consensus phenylalanine residues has been observed in several LRR structures, such as the Nogo receptor and TLR3^32,33^ (Fig. 3). However, the spine formed by the FGxxFN repeat is subtly different in its positioning and the planar stacking arrangement of the highly conserved phenylalanine residues that make up the repetitive hydrophobic core. This constrained arrangement is possible only because the conserved glycine in the FG motif can adopt appropriate PHI/PSI angles in the bottom left quadrant of the Ramachandran plot. To our knowledge, the only other structure containing repeats with a double phenylalanine spine is the recently published structure of LGI1^34^. In this case, the phenylalanine spine is packed in a less restricted manner, and the repeat follows the more conventional packing arrangement with the asparagine ladder being located at the end of the initial β‐strand (Fig. 2a, b). Although the FG-containing peptide in the Crov588 structure is restricted by the packing of neighboring repeats within the protein core, the planar stacking arrangement within the **F**Gxx**F**N motif is in itself interesting. The intermolecular Potential Energy Surface (PES) of a Phe − Phe interaction calculated by MD simulations shows two minima for antiparallel and parallel stacking arrangements^35^. The parallel stacking FF2 minimum shows a distance between the two ring centroids of 4.03 Å and a 5.1° angle between the rings^35^. The internal F-F stacking within the Crov588 FGxxFN repeat is also a parallel stacking mode, albeit with a centroid distance of 3.8 Å and a stacking angle of 34°. The centroid distance between Phe residues in subsequent repeats is 5.6 Å with a stacking angle of 62°.

Crov564 and Crov563 have repeat sequences that are very similar to Crov588 (Fig. 1b). Therefore, we assume that the Crov588 is also representative of these other two family members.

### Surface features of Crov588

The Crov588 repeat shows a preference for histidine or tyrosine at position three, and this feature is conserved in Crov564 and Crov563, but not in Crov527 and Crov539, where this position is replaced with threonine or serine (Fig. 1b). These surface histidine patches proved to be perfect binding sites for the Anderson-Evans polyoxotungstate [TeW_6_O_24_]^6−^ (TEW) cluster used to solve the structure; and indeed, 8 of the 14 cluster sites were found on these histidine patches (Supplementary Figure 1). The concave surface of LRR family proteins is commonly used as a site for protein–protein interactions. In this repeat, however, variable surface features are restricted due to the conserved Histidine ladder and the FG motif. Only residue 5 on the concave surface of each repeat comprises sequence variability and shows a preference for small polar residues S/T or negatively charged E. The most variable residues in the repeat are at positions 8–9 in between the FG and FN elements along the edge of the concave surface. Here, the charged residues EE in repeats 2–3 (Glu^128^, Glu^129^, Glu^150^, Glu^151^) and DD in repeats 11–13 (Asp^326^, Asp^327^, Asp^348^, Asp^349^, Asp^370^, Asp^371^) form two clusters of negative charges along the edge of the concave surface of the Crov588 structure.

### Overall structure of Crov539

**Figure 4:**
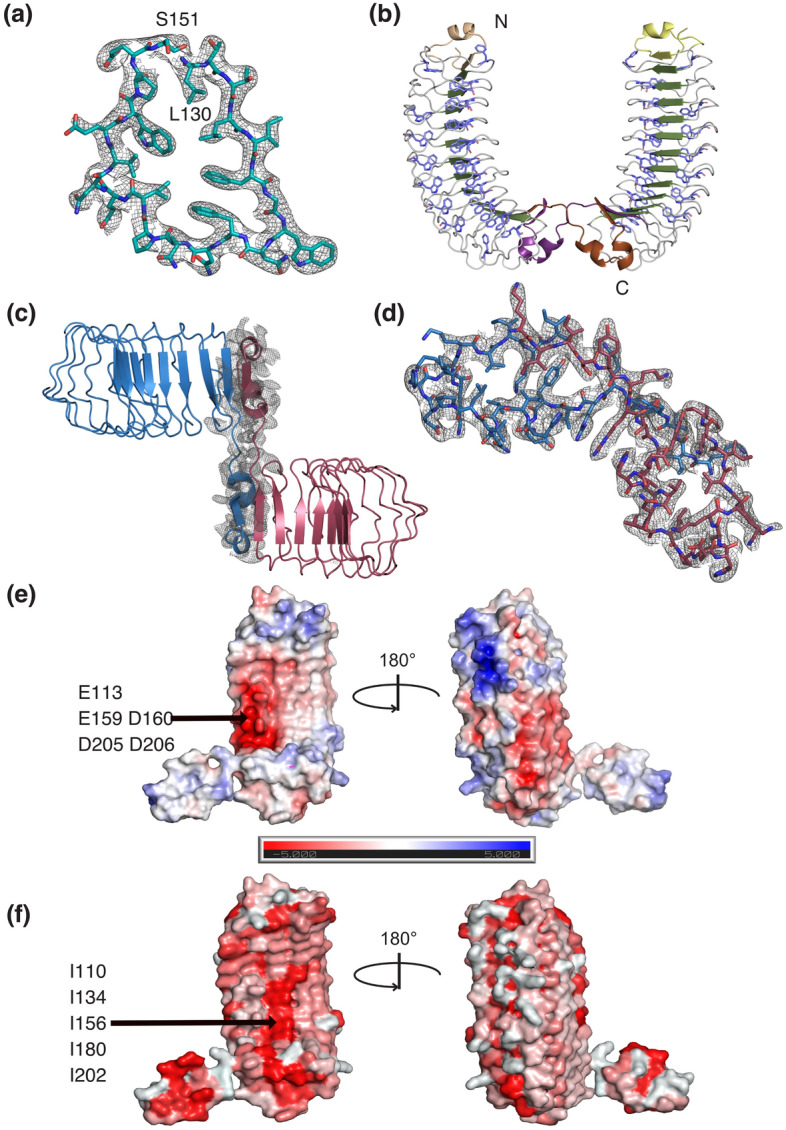
Structure of the Crov539 FGxxFN LRR protein. (**a**) Zoomed view of an individual repeat is presented with 2Fo-Fc density contoured at 1.0σ. (**b**) Ribbon representation of the Crov539 structure. Highlighted are N and C-terminal capping repeats (wheat-purple, yellow–brown), the phenylalanine/tryptophan spine (Slate). (**c**) Representation of the Crov563 dimer with individual chains colored blue and raspberry, 2Fo-Fc density at the dimer interface is contoured at 1.0σ. (**d**) A close-up of the motif swapped dimer region illustrated in sticks with 2Fo-Fc density at the dimer interface is contoured at 1.0σ. (**e**) Charged surface features of the Crov539 structure calculated with APBS, highlighting the pronounced negatively charged patch in the central curvature of the structure. (**f**) Hydrophobic surface features of the Crov539 structure colored according to the Eisenberg hydrophobicity scale with red being most hydrophobic.

**Figure 5:**
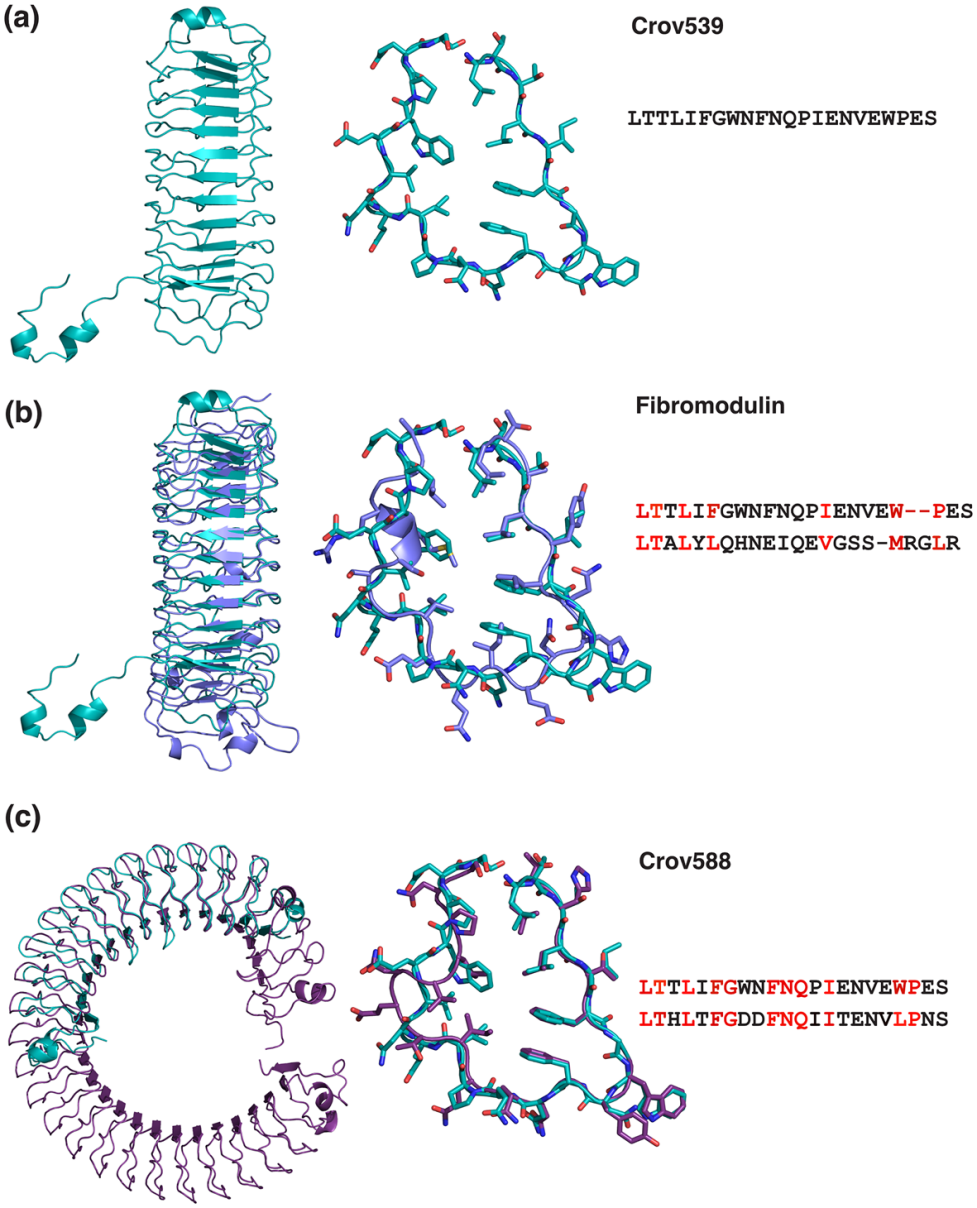
Overlay of structures and aligned LRR motifs for (**a**) Crov539, (**b**) Fibromodulin (5MX0) and (**c**) Crov588 (6NYR).

Crov539 and Crov527 represent the second sub-class of the here analyzed repeat proteins with an LxxLxFGxxFNQPIExVxW/LPxx consensus (Fig. 1b, supplementary sequence file). Differences to Crov588 are the lack of histidine at the N-termini of the repeats and the occurrence of a proline in the middle and of tryptophan near the C-terminus of the repeat. We crystallized Crov539 and solved its structure by molecular replacement using a homology model based upon the Crov588 structure. Crov539 has a short and curved core comprising 12 regularly shaped repeats (Fig. 4). This core is flanked by a compact 15-residue N-terminal capping module. At the C-terminus, a unique domain swapping arrangement occurs where the last repeat(s) (261–290) of the first molecule extends across and caps another molecule to form a dimer. Dimerization is also evident in gelfiltration experiments. To our knowledge, such an arrangement has not been reported for other LRR structures. For both the Crov527 and Crov539 repeats, the packing within the hydrophobic core is altered through a substitution of the LP motif for WP at positions 19–20 (Fig. 1b). This appears to be restricted to those repeat proteins that also contain a restraining proline at position 13. The restriction between prolines 13 and 20 and the inclusion of a bulky tryptophan residue gives rise to a reversal of the hydrophobic core packing at residues 17 and 18. A search of the DALI database^36^ highlighted our Crov588 structure (RMSD 2.1 Å) and the structure of human fibromodulin (5mx0, RMSD 2.9 Å)^37^ as the closest structural homologs. While fibromodulin is of similar size and curvature to Crov539, its repeat sequence and structure show significant differences in hydrophobic core packing (Fig. 5).

### Surface features of Crov539

Similarly, to the Crov588 structure, tightly bound water molecules participate in hydrogen bonding and stabilize the irregular loop structure along the convex surface of the structure.

Unlike Crov588, Crov539 lacks the histidine ladder derived from position 3 of the repeat as this residue is almost exclusively threonine. Residue 5 on the concave surface of the repeat is uncharged and either threonine at the N-terminus of the structure or isoleucine (Ile^110^, Ile^134^, Ile^156^, Ile^180^, Ile^202^) towards the C-terminal region, creating a hydrophobic patch at the center of the lower concave surface (Fig. 4f). Adjacent to this hydrophobic patch at the edge of the concave surface is a negatively charged surface generated from the variable residues (Glu^113^, Glu^159^, Asp^160^, Asp^205^, Asp^206^) at positions 8 and 9 in the repeat, directly following the FG motif (Fig. 4e).

### Analysis of the Crov588 and Crov539 repeat geometry

Given the completely circular architecture and the highly repetitive repeat structure of Crov588, we were intrigued to compare its geometry to other classical LRR proteins. The curvature, twist, and lateral bending angles for each repeat relative to its predecessor can be calculated using the ANGULATOR webserver (http://bragi2.helmholtz-hzi.de/Angulator/)^38^ (Supplementary Figure 2). We used this to compare the Crov588 structure with its structural homologs from the PDB; TLR3 (1ZIW) and YopM (1JL5)^32,39^ as determined from searching the DALI database^36^ (Fig. 3). Additionally, we included the archetypical ribonuclease inhibitor structure (2BNH)^40^. The central diameter of ribonuclease inhibitor is roughly half that of Crov588 at only 20 Å. Therefore, despite the circular structure of Crov588, its cumulative curvature of 343° (12.2° per repeat) is not as pronounced as that of ribonuclease inhibitor with a cumulative curvature of 289° (19.3° per repeat) (Supplementary Figure 2). In comparison, Crov539 illustrated a similar curvature to Crov588 with a cumulative value of 129° (11.7° per repeat).

## Discussion

FNIP repeat proteins are a large and still growing class of leucine-rich repeat (LRR) proteins for which, so far, no experimental structures were available. We now report the first structures, namely those of Crov588 and Crov537. Their perhaps most striking structural feature is a fully buried double phenylalanine spine that originates from their diagnostic FGxxFN motifs. The two repeat proteins are related but show some differences in their repeat consensus (see Fig. 1b) and the packing of their hydrophobic core. The *Cafeteria roenbergensis* virus encodes ~ 70 FNIP repeat proteins (see Supplementary sequence file). The majority of them follow either the Crov588 repeat consensus (**L**xx**L**x**FG**xx**FN**Qx**I**xEN**VLP**xx; bold residues forming the hydrophobic core) or the Crov539 repeat consensus (**L**xx**L**x**FG**xx**FN**x**P**x**I**ENx**WP**xx). The two structures are, therefore, ideal templates for modeling structures of the remaining Crov repeat proteins.

The FNIP LRR protein class was named after the occurrence of the FNx_7_IP motif. The C-terminal IP sub-motif is, however, not conserved but substituted by LP in Crov588 and by either WP or LP in the Crov539 class. The preceding FG dipeptide shows, in fact, better conservation. ‘FGxxFN LRR proteins’ might therefore be a more accurate denomination also because it considers the structural relevance of the FGxxFN motif.

We searched for further FGxxFN LRR proteins using hidden Markov modeling tools (https://www.ebi.ac.uk/Tools/hmmer/) and the Crov588 sequence as an input. This returned, amongst others, 557 sequences from *Dictyostelium* (mostly with C-terminal “LP” sub-motifs), 104 sequences from the brown alga Endocarpus (mostly with C-terminal WP sub-motifs), and 51 from Megavirus chiliensis (predominantly with C-terminal IP sub-motifs). Recently, 82 Megavirus baoshan proteins have been reported that feature a similar but shorter (21 instead of 22 residues) VxxLxFGxxFNQxIxxxIPxx repeat consensus^23^.

Obviously, these species have exploited the variability within a single FGxxFN repeat to evolve a family of proteins that may recognize a large number of potential ligand molecules. They are not quite as diverse as the Variable Lymphocyte Receptors (VLRs) of jawless fish, which are generated by somatic gene rearrangements and have an antibody-like adaptive immune function^41^. Nevertheless, it is astounding how large the FGxxFN LRR repertoire of individual species can be.

So far, little is known about the functions of FGxxFN LRR proteins—though their diversity suggests also functional diversity and possibly cross-species differences. For example, many of the Megavirus baoshan FGxxFN LRR domains are fused to an N-terminal F-box domain, indicating that they function in the ubiquitin–proteasome pathway^23^ and that the repeat domain might select proteins for degradation. In contrast, numerous FGxxFN LRR domains from *Polysphondylium pallidum* are linked to kinase domains and thus might confer specificity in protein phosphorylation. The Cafeteria repeat proteins, on the other hand, appear not associated with any enzymatic activities. Instead, they might have structural roles in the cytoplasmic virus replication factories^42^, or bind cellular factors as stoichiometric agonists or antagonists to subvert the host’s anti-viral response.

The Crov588 structure is, to our knowledge, the first structure of a naturally occurring closed circle of LRR motifs. This was previously only shown for a designed LRR structure with 20 LRRs created to explore engineered binding scaffolds^43^. The concave (inner) surface of LRR family proteins is commonly used as a site for protein–protein interactions^30^; although in some cases, exterior surfaces are used as the predominant ligand binding site, as in the case for TLR3 and CD14^44,45^. FGxxFN LRR proteins probably also follow a concave surface-binding mode since positions 8–9 (at the FGxxFN motif) on the edge of the repeat are the most variable ones.

Our initial reason for studying the Crov repeat proteins was to investigate the packing arrangement of phenylalanine residues with respect to the conserved FG dipeptide motif within the repeat sequence. Similar motifs are commonly found in the intrinsically disordered repeat regions of nucleoporins. The interactions of the different types of FG motifs (FxFG, PxFG, SAFG, GLFG, and FG)^13,46^ within the nuclear pore are dynamic and likely to include mixtures of stacking arrangements such as staggered stacking, parallel in plane, tilted, edge-ring-face, and cogwheel, which have all been described in bioinformatic analyses of protein structures^47–50^. More recently, the structure of a GFGNFGTS peptide trapped in a cross-β arrangement has been determined^51^. Here the peptide stacks as segments of kinked β-sheets that pair into protofilaments with layers of phenylalanine interactions. The layering within this structure restrains the distance between Cα atoms between the Phe residues because of the β-sheet formation, while the shortest van der Waals contact is 3.5 Å. The phenylalanine residues stack here in a parallel staggered arrangement with an angle of 48° between the rings and a centroid distance of 4.8 Å. In the same way, we can consider the double phenylalanine spine of consecutive FGxxFN repeats simply as another parallel stacking of FG motifs, albeit within the hydrophobic core of a soluble protein and not an assembled fiber. At present, it is unclear how representative this arrangement for cohesive interactions within intrinsically disordered FG repeat domains is; however, the Crov structures illustrate one possible mode of interaction.

## Methods

### Protein expression and purification

We expressed and purified codon-optimized (for E*. coli*) versions of Crov527, Crov539, Crov563, Crov564, and Crov588 as His_14_-bdSUMO or H14-zz-bdSumo-Crov fusion proteins. The His-tagged proteins were purified using Ni(II) chelate beads, using extensive washing in buffer A (50 mM Tris pH8.0, 300 mM NaCl, 10 mM Imidazole, 1 mM DTT), high salt buffer B (50 mM Tris pH8.0, 1 M NaCl, 10 mM Imidazole, 1 mM DTT), ATP wash buffer C (10 mM Tris pH8.0, 100 mM KCl, 10 mM Imidazole, 5 mM MgCl2, 1.5 mM ATP, 1 mM DTT), low salt buffer D (10% Glycerol, 10 mM Imidazole, 1 mM DTT). Elution was by on-column-tag-cleavage with a SUMO tag-cleaving protease in elution buffer E (50 mM Tris pH8.0, 300 mM NaCl, 5 mM Imidazole, 10% glycerol, 1 mM DTT) as described before^52,53^. We were able to crystallize the Crov539, Crov588 and Crov564 proteins. However, crystals of Crov564 observed in HTS trials were not reproducible.

### Differential scanning fluorometry (DSF/Thermofluor)

Proteins were diluted to 1 mg/ml in 20 μl 50 mM Tris/HCl pH 8.0, 300 mM NaCl, 1 × SYPRO Orange (Life Technologies). Experiments were performed in a Hard-Shell® 96-well plate (Bio-Rad) sealed with transparent MicroSeal “B” Seal (Bio-Rad), using the CFX96 Real-Time System (C1000 Thermal Cycler, Bio-Rad). The samples were incubated for 5 min at 20 °C before the temperature was gradually increased to 95 °C with 1-K increments and 45 s for each incubation step. At the end of each step, the SYPRO Orange fluorescence was measured using the HEX channel. Non-linear fitting of truncated fluorescence data and Tm value determination was carried out using GraphPad Prism.

### Crystallization and structure determination of Crov588

Several initial crystallization conditions for Crov588 were identified by high-throughput screening with 100 nL + 100 nL drop volumes using a Crystal Gryphon robot (Art Robbins Instruments) and commercial grid and sparse matrix screens (Hampton Research, Qiagen, Jena Biosciences, Molecular Dimensions). The best crystals were reproduced and grown via hanging drop vapor diffusion by equilibrating a 1.5 μl drop of protein with 1.5 μl of reservoir solution. Final crystallization conditions were 0.1 M MES pH 6.0, 20% PEG3000, 0.1 M MgCl_2_. Crystals were cryoprotected gradually by soaking in reservoir solution containing increasing amounts of ethylene glycol up to 25–30% for several minutes, before being mounted directly from the drop in a Mitogen loop and flash frozen in liquid nitrogen. For the Anderson − Evans polyoxotungstate [TeW_6_O_24_]^6−^ (TEW) derivative, a final concentration of 1 mM was added to the cryoprotectant solution from a 100 mM stock (Jena Biosciences).

Diffraction data were collected at 1 Å wavelength for native data and 1.2108 Å for the Anderson tungstate derivative using a PILATUS 6M (Dectris) detector at the PXII beamline (Swiss Light Source, Villigen, Switzerland) and all data processed with XDS^54^ (for statistics see Table 1). Native Crov588 crystals showed good diffraction to 2.4 Å and belong to the space group P1211 with two molecules in the asymmetric unit and 63% solvent. In contrast, the TEW soaked crystals showed weaker diffraction to 3.0 Å resolution; soaking also appeared to alter the symmetry of the crystals to be space group P2_1_2_1_2_1_. The structure was initially solved in this space group with 1 molecule in the asymmetric unit and 63% solvent using the data from two TEW soaked crystals. Initial phases were obtained by W-SAD using the Phaser-SAD routine running Hyss^55^ which identified several individual heavy atom sites. Tungstate clusters were positioned manually and recycled again using the Phaser-SAD routine, this procedure was repeated several times before 4 clusters could be accurately positioned on the concave surface of the Crov588 repeat.

After density modification with Resolve^56,57^, the map was easily interpretable and the central 60% portion of the repeat sequence could be automatically traced using Buccaneer^58^. This partial model was then used to solve the higher resolution native dataset by molecular replacement using Molrep^58^. The remainder of the structure was built by multiple cycles of manual rebuilding in COOT^59^ with refinement using both REFMAC5^60^ and Phenix^56^. Final refinement of the structure, converged with *R*cryst = 22% and *R*free = 25%. The final Crov588 model was then used to refine against the W-SAD data and at this stage it became clear that the true space group of the tungsten data was also P1211, with a β-angle of 90.13 compared to the β-angle of 94 for the native data. The structure was then resolved by molecular replacement and re-refined. Additional residual tungsten sites were also visible at this stage, final refinement was checked for both structures using the PDB-REDO server^61^ (for statistics see Table 1).

### Crystallization and structure determination of Crov539

Crystallization conditions were found after setting up a single commercial screen, namely the Hampton Research PEGRx screen. Clusters of thin plate crystals were grown via hanging drop vapor diffusion by equilibrating a 1.5 μl drop of protein with 1.5 μl of reservoir solution (PEGRX2 42) containing 0.1 M Bis-TRIS pH 6.5, 10% PEG10,000, 0.2 M Potassium Sodium Tartrate tetrahydrate. Crystals were cryoprotected gradually by soaking in reservoir solution containing increasing amounts of ethylene glycol up to 25% for several minutes, before being mounted directly from the drop in a Mitogen loop and flash frozen in liquid nitrogen.

Diffraction data were collected at 1 Å wavelength using a EIGER 2 16 M detector at the PXII beamline (Swiss Light Source, Villigen, Switzerland) and all data processed with XDS^54^ (for statistics see Table 1). Native Crov539 crystals showed good diffraction to 2.73 Å and belong to the space group P22_1_2_1_ with 6 molecules in the asymmetric unit and 63% solvent.

The Crov539 structure was solved by molecular replacement with Phaser^62^ using a homology model generated with Modeller^63^ from the previously solved Crov588 structure. The structure required multiple cycles of manual rebuilding in COOT^59^ with refinement using both REFMAC5^60^ and Phenix^56^. Final refinement of the structure, converged with *R*cryst = 21% and *R*free = 26%.

### Structural and bioinformatic analysis

Structural alignments were carried out in coot^59^, centroid distances and angles were calculated using pseudo atoms and all figures prepared using Pymol (Schrödinger).

## Supplementary Information

Supplementary Information.

## Data Availability

GenBank accession numbers for the Crov FGxxFN LRR proteins are ADO67561.1 (Crov527), ADO67573.1 (Crov539), QCH59643.1 (Crov588). The crystal structures have been deposited in the protein data bank with the following accession codes; Crov588 (6NYR), Anderson–Evans polyoxotungstate [TeW_6_O_24_]^6−^ (TEW) derivative (6NYS) and Crov539 (7ZAL).

## Abbreviations

LRR: Leucine-rich repeat
Crov: *Cafeteria roenbergensis* Virus
