# Supplementary material for: Crystal structures of FNIP/FGxxFN motif-containing leucine-rich repeat proteins
Source: Sci Rep. 2022 Sep 30;12:16430. doi: 10.1038/s41598-022-20758-8 (PMC9525666; doi:10.1038/s41598-022-20758-8)
Supplement: Supplementary file 1 — Supplementary Information. [file 41598_2022_20758_MOESM1_ESM.pdf]

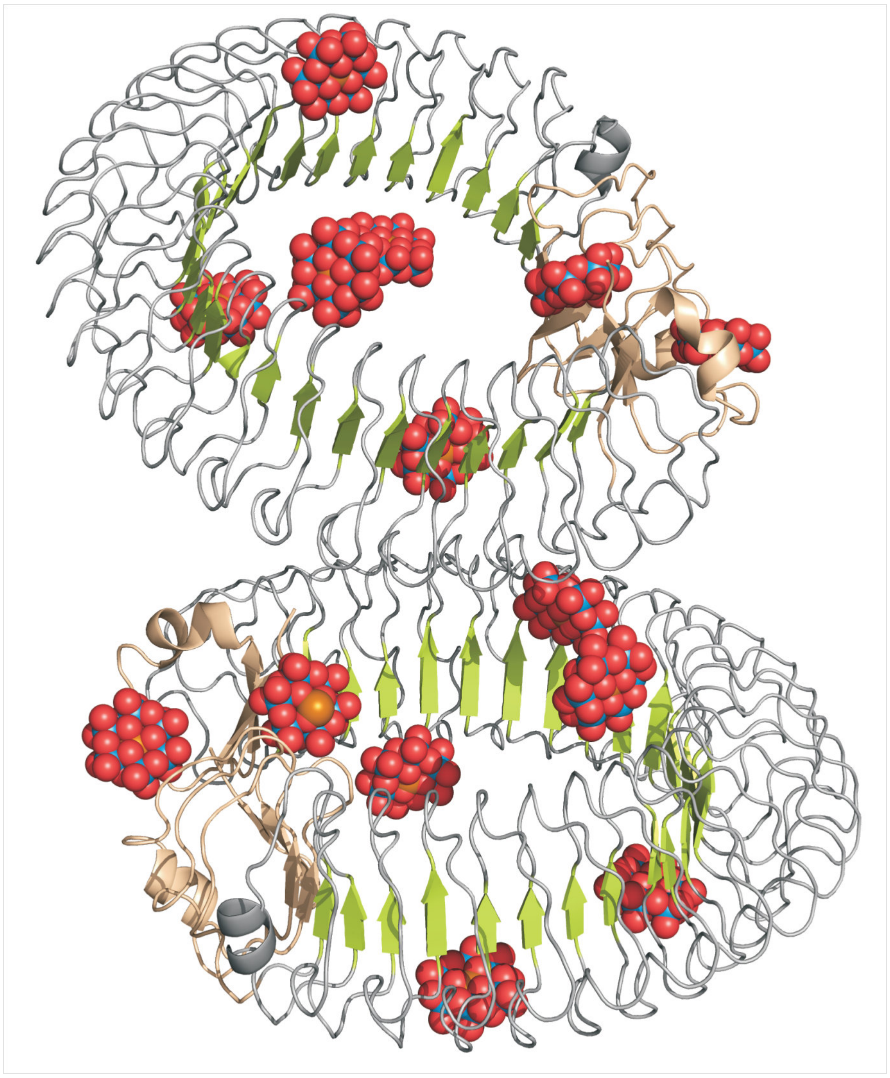

Supplementary Figure 1.

Depiction of the Anderson−Evans polyoxotungstate $[TeW_6O_{24}]^{6-}$ (TEW) binding sites within the derivative structure. Clusters are shown in spheres with 14 clusters per ASU.

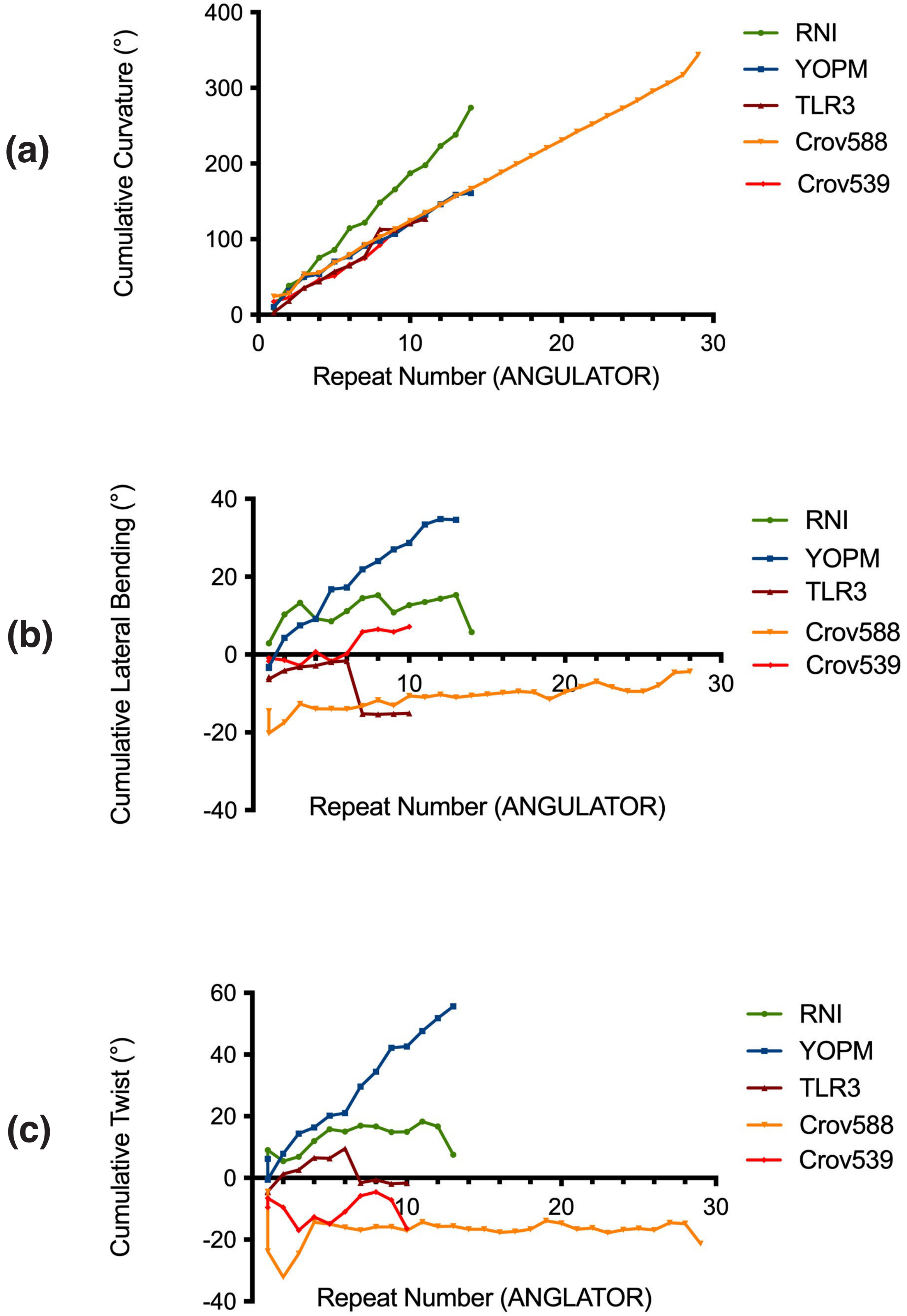

Supplementary Figure 2.

Plot of (a) curvature, (b) twist, and (c) lateral bending angles for each repeat relative to its predecessor as calculated using the ANGULATOR webserver (http://bragi2.helmholtz-hzi.de/Angulator/). Depicted are the structures for Ribonuclease inhibitor (RNI, 2BNH), *Yersinia* outer protein M (YopM, 1JL5), Toll-like Receptor 3 (TLR3), Crov588 and Crov539.

Supplementary sequence file.

The sequences of FGxxFN/ FNIP repeat-containing proteins from Cafeteria roenbergensis virus (Crov) colored to highlight the repeat conservation and two distinct subclasses of Crov FGxxFN repeats.

```
>Crov588
MEKYTIKETILTFNNEFNDPLDKYYKILSNPK
IDTIEFGEKFNQEIDHLIPSN
IKVIKFGWTSEFNKDVNFLTES
LTEIYYGIYKNHSLEELQNLPKS
LLKLKLGDVFNQEIVENVLPGG
LTHLTFGEEFNQKIVENVLPGG
LTHLTFGEEFNQKIVENVLPNS
LTHLSFGDCFNQKITENVLPNS
LTYLEFGRNFNQKITENVLPNS
LTHLTFGWYFNQQITENVLPNS
LTYLEFGRNFNQQITENVLPNS
LTYLEFGRNFNQQITENVLPNS
LTHITFGNNFNQIITENVLPNS
LTHLTFGNNFNQIITENVLPNS
LTHLTFGDDFNQIITENVLPNS
LTHLTFGDDFNQIITENVLPNS
LTHLTFGDDFNQIITENVLPNS
LVHLSFGCEFNQEIAEKVLPNS
LTYLELGHNFNQKIIENVLPNG
LVHLSFGCKFNQEIVENVLPDS
LTHLSFGHCFNQKITENVLPNS
LTYLELGHNFNQKIIENVLPDR
LTYLELGHDFNQKIMENVLPNS
LTHLIFGTSFNQNLTENVLPNS
LTHLTFGTCFNQKIIENVLPNS
LTHLEFGPKFNQKITENVLPNS
LTHLTFGTSFNQKITENVLPNG
LTYLTFGLRFNQKITENVLPCS
LTHLTFGWYFNQELTENVLPDT
LKVLKIYYGNKDIILKNIDTSKIKFKIEYFNKN

>Crov032
MRMYVFQMELFDALNLLGNGSNKFDFTFTFKNGTTLFYNSELKSWQISKYIKNNKLELILKIIRIIINGINLSNGG
CELEYVP
VAYYNYKTDTTIELWRLQLPNS
LTHLTFGFKFYQEITENVLPTN
LKVLKIYYGNKNKDIILKNIDTSKIKFKIEYFK

>Crov031
MKEYTIK
NTILTFNDEFNEPLDKYYKILSNPK
IDTIEFGEKFNQEIDHLIPSN
IKVIKFEPNSRFNKDVNFLTES
LTEIYYGIYKNHSLEELQNLPKS
LLKLKLGDVFNQEIVENVLPGG
LTHLTFGEEFNQKIVENVLPNS
LTHLSFGDCFNQKITENVLPNS
LTYLEFGRNFNQKITENVLPNS
LTHLTFGWYFNQEIVENVLPNS
LTRLSFGYCFNQKITENVLPNS
LTHLIFGYIFNQKIIENVLPNS
LTYLELGHNFNQRIIENVLPNS
LTHLSFGYEFNQEIVENVLPGG
LTHLTFGGYFYQKITKNVLPSS
LTHLTFENNFNQEITENVLSNS
LTYLIHLPYN
```

>Crov608
MEEYIIE
NNILRFNNEFNDPLDKYYKVLSYPE
IDTIEFGDDFNQEIDHLIPSN
IKVIKFEPNSRFNKDVNFLTES
ITEIYYGIYKNHSLEELQNLPKS
LLKLRLGSKFNQQITENVLPNS
LTHLSFGWRFNQKITKNVLPNS
LTYLTFGSNFNQEITENVLPNS
LTYLTFGTNFNQEITENVLPNS
LTHLDFGYDFNQKIIENILPNS
LTHLTFGYNFNQKIIENVLPNS
LTHISFGDCFNQKITENVLPNS
LTHLTFGLHFNQQITENVLPDG
LTHISFGYCFNQKITENVLPCS
LTHLTFGYNFNQKIIENVLPNS
LTYLDLGEDFNQKLTENVLPNS
LSHLSFGEKFNQEITENVLPNS
LTHLTFGEKFNQEITENVLPNS
LTYLELGEDFNQKLTENVLPNS
LSHLSFGYKFNQEIVENVLPNS
LQALKIHHRNRDIILKNIDTSKITFEIECFY

>Crov607
MFLLKINLLFNYT
LTYLTFGYKFNQKITKNVLPSR
LTHLSFGYKFYQEITENVLPNS
LTHLTFGNCFNQKITDNILPNS
LTHLTFGTLFNQEITENVLPCS
LTHLTFKRNFKQELTSNILPDN
LKVLEIHYGNKNKDIILKNIDTSKIKFKIE

>Crov606
MEEYIIKNNILTFNYEFNEPLDKYYKVLSYPE
IDTIEFGEDFNQEIDHLIPSN
IKIIKFGWDSKFNKDVNFLTESL
TEIYYGKYKNHSLEELLNLPKS
LLKLQLGDVFNHEITENVLPGG
LTHLTFGKEFNQKIVENVLPGG
LTHLTFGKEFNQKIVENVLPDG
LTHLTFGWYFNQQITENFLPNS
LTHLIFGFVFDQKLTENVLPNS
LTHLTFGYNFNQKIVENVLPDG
LTHLTFGYNFNQKIIENVLPNS
LTHLTFERYFSQKITENVLPNS
LTHLTFYKEFNQEIVENVLPNN
LTHLTFGYYFNQEIKKNVLPNS
LTHLTFGYYFNQEIKKNVLPGG
LTHLTFVGDFNQKIVENVLPSG
LTHLDFGTDFNQEITENVLPNS
LTHLTFGDKFNQIITENVLPNS
LTHLTFGDKFNQIITENVLPNS
LTHLTFGEKFNQEITENVLPCG
LTHLTFGWYFNQQITENVLPNN
LKVLKIYYLNKDIILKNIDTSKIKFKIEYF

>Crov605
MEEYIIKNNILTFNYEFNEPLDKYYKILSYPE
IDTIEFEYKFNQEIDHLIPSN
VKVIKFGPYSKFNKNVNFLPES
ITEIYYGPNKNHSLEELQNLPKS
LLKLRLGGIFNHKITENVLPNS
LTYLAFGWRFNQKITKNVLPNS
LTYLTFGSNFNQEITENVLPNS
LTHLTFGYYFNQEITVNILPNS
ITYLTFGWNFNQKITENVLPNG
LTHLTFGEEFNQKIVENVLPNS
LTHLSFGDGFNQKITENVLPNS
LTYLEFGRNFNQKITENVLPNS
LTYLEFGRNFNQKITKNVLPNS
LTHLTFGWYFNQQITENVLPKS
LTHLIFGYVFNQKLTENVLPNS
LTHLAFGWCFNQKITKNVLPNS
LTHLIFGYVFNQKLTENVLPNS
LTHLSFGYYFNQEITENVLPNS
LTHLSIGCWFNQKITENVLPTG
LTHLTFERNFKQELTLNILPDN
LKVLKIYYGNGNNYKDIILKNIDTSKIKFKIEYFK

>Crov604
MEEYIIENNILTFNNEFNEPLDKYYKVLSTPE
IDTIEFGDDFNQEIDHLIPSN
IKVIKFGPNSEFNKDVNFLTES
LTEIYYGIYKNHSLEELQNLPKS
LLKLRLGGHLNQKITENVLPNS
LTHLAFGWRFNQKITKNVLPNS
LTHLTLGNSIVDSYFNQKITDNILPNS
LTHLTFGSRFNQKITKNVLPDG
LTHLTFGWHFNQQITENVLPGG
LTHLTFGYYFNQEIKKNVLPCS
LTHLIFGYWFNQKLTENVLPNS
LTHLTFGDDFNQIITENVLPNS
LTHLTFGWHFNIQITENVLPNS
LTHLSFGCWFNQKITKNVLPSS
LTYLTFGRDFNQKITENVLPSS
LTHLTFGNNFNRKITENVLPDN
LKVLKIYYGNKDIILKNIDTSKIKFKIEYFK

>Crov603
MEEYIIENNILRFNDEFNEPLDKYYKVLSYPE
IDTIEFGRNFNQEIDHLIPFN
IKVIKFEPNSRFNKDVNFLTES
ITEIYYGIYKNHSLEELQNLPKS
LLKLRLGSKFNQQITENVLPDG
LTHLTFGKEFNQKIVENVLPTS
LTHLTFSYCFNQKIVENVLPGG
LTHLTFGATFNQKITDNILPNS
LTHLTFGGRFNQKITKNVLPSS
LTHLSFGHKFNQKIIENVLPTS
LTHLTFENNFNQKITENVLPGG
LTHLTFGKEFNQQIVENVLPDG
LTHLTFGWYFNQQITENVLPNS
LTYLEFGHNFNQQITENVLPNS
LTYLKFGRDFNQIITENVLPNS
LTHLTVGFGLSCKESTLNILPKN
LQVLQLINIDKDIILKNIDTSKIKFKIQYFK

>Crov602
MEEYIIENNILRFNNEFNEPLDKYYKVLSTPE
IDTIEFGYKFNQEIDHLIPSN
VKVIKFGQYSKFNKNVNFLPES
ITEIYYGPNKNHSLEELQNLPKS
LLKLRLGGIFNHKITENVLFNS
LTHLAFGWRFNQKITKNVLPNS
LTYLTFGSNFNQEITENVLPNS
LTHLTFGYYFNQEITVNILPNS
ITYLTFGWNFNQKITENVLPDG
LTHLTFGKYFNQKITENVLPNS
LTHLTFGYNFNQKITENVLPNS
LTHLTFGANFNQKITDNILPNS
LTHLTFGLCFYQKITKNVLPSS
LTHLSFGHKFNQKIIENVLPNS
LTHLTFENNFNQKITENVLPNS
LTHLTFGDDFNQIITENVLPDG
LTHLTFGWNFNQQITENVLPNS
LTHLTFGGRFNQKITENVLPDG
LTHLTFGGRFNQKITDNILPNS
LTHLTFGYDFNQKITENVLPNS
LTHLTFGRHFNQKITENVLPNS
LTHLSIGCWFNQKITENVLPTS
LTHLTFEMNFKQETLNILPDN
LKVLKIYYKNKDIILKNIDTSKIKFKIEYFK

>Crov601
MEEYIIENNILTFNNEFNEPLDKYYKVLSTPE
IDTIEFGNDFNQEIDHLIPSN
IKVIKFGHDSEFNKDVNFLTES
LTEIYYGIYKNHSLEELQNLPKS
LLKLRLGHLNQKITENVLPGG
LTHLTFGYYFNQEIKKNVLPCS
LTHLIFGYWFNQKLTENVLPNS
LTHLTFGWHFNIQITENVLPNS
LTHLSFGCWFNQKITKNVLPSS
LTYLTFGRDFNQNLTENILPTS
LTHLTFSYCFNQKITENILPNS
LTHLTFGSRFNQKITENVLPDG
LTHLTFGWHFNQQITENVLPDG
LTHLTCGWHFNQQITENVLPNS
LTHLTFGSRFNRKIVENVLPNS
LTHLTFGDSFNQIITENVLPNS
LTHLTFGDNFNQIITENVLPNS
LTHLTFGNNFNQKITKNVLSNS
LTHLSFGCWFNQKITENVLPTS
LTHLTFERNFKQELTLNILPDN
LKVLEIYYQNKDEILKNIDTTKIKFEIEYLIKN

>Crov600
MEEYIIENNILRFNNEFNEPLDKYYKVLSNPE
IDTIEFGRNFNQEIDHLIPSN
IKVIKFGWNSEFNKDINFLTES
LTEIYYGKYKNHSLEELQNLPQS
LLKLKLGDVFNHEITENVLPGG
LTHLTFGKCFNQKITENVLPGG
LTHLTFGWYFNQQITKNVLPNS
LTHLSFGYKFNQEITENVLPNS
LTHLSFGDCFNQKITENVLPNS
LTHLTFGWHFNQQITENVLPNS
LTHLSFGYEFNQKITENVLPNS
LTHLSFGCWFNQKITENVLPTS
LTHLTFERNFKQETLNILPDN
LKVLKIYYKNKDIILKNIDTSKIKFKIEYF

>Crov599
MEEYIIENNILRFNNEFNEPLDKYYKVLSTPE
IDTIEFGENFNQEIDYLIPSN
IKVIKFEPNSRFNKDVNFLTES
LTEIYYGTYKNHSLEELQNLPKS
LLKLRLGHLNQKITENVLPGG
LTHLTFGYYFNQQITENVLPDG
LTHLTFGWHFNQQITENVLPDG
LTHLTFGWHFNQQITENVLPNS
LTHLIFGYMFNQKLTENVLPNS
LTHLTFGYNFNQKIIENVLPS
LTHLTFGKYFNQKITENVLPNS
LTHLTFGYNFNQKIIENVLPNS
LTHLTFGGRFNQKITENVLPNS
LTHLTFGLCFYQKITKNVLPGG
LTHLTFGEEFNQKIVENVLPDG
LTHLTFGWDFNQQITENVLPNS
LTYLQFGRNFNQQITENVLPNS
LTYLKFGRNFNQQITENVLPNS
LTHLTVGYGLSYKESTLNILPKN
LQVLQLINIDKDIILKNIDTSKIKFKIQYFK

>Crov598
MEEYIIENNILRFNNEFNEPLDKYYKVLSTPE
IDTIEFGYKFNQEIDHLIPSN
IKVIKFECRSKFNKDVDFLTES
LTEIYYGSNKNHSLEELQNLPKS
LLKLRLGSKFNQQITENVLPTS
LTHLTFSYCFNQKIVENVLPGG
LTHLTFGATFNQKITDNILPNS
LTHLTFGGGFNQKITKNVLPSS
LTHLSFGHKFNQKIIENVLPNS
LTHLTFENNFNQKITENVLPNS
LTHLTFGDDFNQIITENVLPGG
LTHLTFGWNFNQQITENVLPNS
LTHLTFGGRFNQKITENVLPDG
LTHLTFGGRFNQKITDNILPNS
LTHLTFGYDFNQKITENVLPNS
LTHLTFGRHFNQKITENVLPNS
LTHLSISCWFNQKITENVLPTS
LTHLTFEMNFKQELTLNILPDN
LKVLKIYYGNKDIILKNIDTSKIKFKIEYFK

>Crov597
MKEYTIKKTILTFNNAFNEPLDKYYKVLSNPE
IDTIEFGENFNQEIDHLIPSN
IKVIEFGWNTEFNKDVNFLTES
LTEIYYGKYKNHSLEELQNLPKS
LLKLKLGDVFNQEIVENVLPSG
LTHLTFGEEFNQKIVENVLPGG
LTHLTFGKEFNQQIT

>Crov595
MEEYIIENNILRFNDEFNEPLDKYYKVLSNPE
IDTIEFGYKFNQEIDHLIPSN
IKVIKFGHDSEFYKDVNFLTES
LTEIYYGKYKNHSLEELLNLPKS
LLKLQLGHLNQKITENVLPGG
LTHLTFGCNFNQQITENVLPDG
LTHLTFGWHFNQQITENVLPDG
LTHLTFGWHFNQQITENVLPDG
LTHLIFGYMFNQKLTENVLPNS
LTHLTFGYNFNQNLTENVLPTS
LTHLTFSYCFNQKITENILPNS
LTHLTFGGRFNQKITENVLPGG
LTHLTFGKYFNQKITENVLPNS
LTHLTFGRYFNQKITENVLPNS
LTHLTFDKDFNQQITENVLPYS
LTHLTFGKDFNHKITENVLPNS
LTYLTFGTNFNQKITDNILPNS
LTHLSFGCWFNQKITENVLPNS
LTYLTFGATFNQKITDNILPNS
LTHLTFGDRFNQKITENVLPNS
LTHLDFGTDFNQEITENVLPDD
LTHLTFGWDFNQQITENVLPNS
LTYLTFGLTFNQKITENVLPTS
LTHLTFGLRFNQKITENVLPSS
LTHLTFGNNFNQKITKNVLSNS
LTHLTFGDDFNQIITENVVEIKIEF

>Crov594
MKEYIIENNILRFNNEFNEQLDKYYKVLSYPE
IDTIEFGDDFNQEIEHLIPSN
IKVIKFEPNSRFNKDVNFLTES
ITEIYYGIYKNHSLEELQNLPKS
LLKLRLGSKFNQQITENVLPNS
LTHLSFGWRFNQKITKNVLPNS
LTYLTFGSNFNQEIKKNVLPCS
LTHLIFGYWFNQKLTENVLPNS
LTHLTFGDDFNQIITENVLPNS
LTHLSFGCWFNQKITKNVLSSS
LTYLTFGRDFNQNLTENVLPTS
LTHLTFGNNFNQKITKNVLPSS
LTHLIFGYMFNQKLTENVLPSS
LTHLTFGTSFNQKITENVLPNS
LTYLTFGLRFNQKITENVLPCS
LTHLTFGYEFNQELTENVLPNS
LKVLKIYYVNKNIILKNIDTSKIKFKIEYF

>Crov593
MEEYIIENNILRFNDAFNEPLDKYYKVLSNPE
IDTIEFGEEFNQEIDYLIPSN
VKVIKFGQYSKFNKDVNFLPES
IKEIYHGFGENHSLEELQNLPKS
LLKLKLGDVFNHEIVENVLPNS
LTYLEFGRDFNHEITENVLPGG
LTHLTFGYWFNQKLTENVLPSS
LTHLTFGFYFNQKLTENVLPNS
LTHVFFSYMFNQKITENVLPNS
LTYLTFGLRFNHKITENVLPCS
LTHLTFGWDFNQELTENVLPNS
LKVLKIYYGNKDIILKNIDTSKIKFKIEYFK

>Crov592
MEKYTIKETILTFNNEFNEPLDKYYKILSNPK
IDTIEFGEKFNQEIDHLIPSN
IKVIKFGWTSEFNKDVNFLTES
LTEIYYGIYKNHNTEELQNLPKS
LLKLKLGDVFNHEIVENVLPNS
LTHLEFGHNFNHEITENVLPGG
LTHLTFGEEFNQKIVENVLPNS
LTHLSFGDGFNQKITENVLPNS
LTYLEFGRNFNQKITENVLPNS
LTYLEFGRNFNQKITKNVLPNS
LTHLTFGWYFNQQITENVLPKS
LTHLIFGYVFNQKLTENVLPNS
LTHLAFGWCFNQKITKNVLPNS
LTHLIFGYVFNQKLTENVLPNS
LTHLSFGYYFNQEIIKNILPNS
LTHLTFGKKFNQQITENVLPNS
LTHLTFGNNFNQKLTLNILPDN
LKVLEIYCGNKNKDIILKNIDTSKIKFKIEYFK

>Crov591
MKEYIIENNILRFNDEFNEPLDKYYKVLSTPE
IDTIEFGRNFNQEIDHLIPSN
IKVIKFEPYSRFNKDVNFLPES
IKEIYHGFGKNHSLEELQNLPKS
LLKLKLGDVFDHEITENVLPNS
LTYLEFGRDFNHEITENVLPGG
LTHLTFGFYFNQEIIRNVLPKS
LTHLIFGYWFNQKLTENVLPSS
LTHLTFGFYFNQEIIRNVVPKT
LTPLIFNYLFNQKITENVLPNS
LTHLTFGNNFNQKLTLNILPDN
LKVLEIYCGNKNKDIILKNIDTSKIKFKIEYFK

>Crov590
MKEYIIENNILTFNDEFNEPLDKYYKVLSTPE
IDTIEFGYKFNQEIDHLIPSN
IKTIKFECWSQFNKDVNFLTES
LTEIYYGIYKNHSLEELQNLPKS
LLKLKLGDVFDHEITENVLPGG
LTHLTFGCNFNQQITENVLPKS
LTHLIFGYEFNQKLTENVLPGG
LTHLTFGWYFNQKLTENVLPNS
LTHLTFGTCFNQKLTENVLPNS
LTHLEFGPKFNQKITENVLPNS
LTHLTFGTSFNQKITENVLPNS
LTYLTFGLRFNQKITENVLPCS
LTHLTFGWYFNQELTENVLPNS
LKVLKIYYVNKDIILKNIDTSKIKFKIEYFK

>Crov589
MEEYIIENNILRFNNEFNEPLDKYYKVLSTPE
IDTIEFGRNFNQEIDHLIPSN
IKTIKFECWSQFNKDVNFLTES
LTEIYYGIYKNHSLEELQNLPKS
LLKLKLGDVFKHEITENVLPGG
LTHLTFGCNFNQQITENVLPKS
LTHLIFGYEFNQKLTENVLPNS
LTHLTFGTCFNQKLTENVLPNS
LTHLTFGTCFNQKLTENVLPNS
LTHLTFGDDFNQIITENVLPNS
LTHLTFGWQFNIQITENVLPNS
LTHLSFGYEFNQKITENVLPNS
LTHLSFGCWFNQKITENVLPTS
LTHLTFERNFKQELTLNILPDN
LKVLKIYYKNKDIILKNIDTSKIKFKIEYFK

>Crov587
MKEYIIENNILRFNDAFNEPLDKYYKVLSNPE
IDTIEFGENFKQEIDHLIPSN
VKVIKFGQYSKFNKDVNFLPES
IKEIYHGFGKNHSLEELQNLPKS
LLKLKLGDVFNHEITENVLPNS
LTYLEFGRNFNHEITENVLPNS
LTYLEFGRNFNHEITENVLPGG
LTHLTFGWYFNQKLTENVLPNS
LTHLTFGSSFNQKITENVLPNS
LTYLTFGLRFNQKLTENVLPNS
LTHLSFYYCFNQKITENVLPNS
LTHFAFGWCFNQKITENVLPNS
LTHLTFGGHFNQQITENVLPDN
LKVLKIYYGNKYIILKNIDTSKIKFEIEYFN

>Crov586
MEEYIIENNILRFNNKFNEPLDKYYKILSYPE
IDTIEFGYKFNQEIDHLIPSN
VKVIKFGWNSEFNKDINFLTES
LTEIYYGKYKNHSLEELQNLPQS
LLKLELGDVFDHEITENVLPGG
LTHLTFGKCFNQKITENVLPGG
LTHLIFGECFNQKITENVLPGG
LTHLTFGWYFNQKLTENVLPNS
LTHLTFGICFNQKIIENVLPNS
LTHLTFGWHFNQQITENVLPNS
LTHLSFGCWFNQKITENVLPNS
LTHLAFGWCFNQKITKNVLPNS
LTHLTFGNDFNQKITENVLPNS
LTYLEFGRNFNQEIVENVLPKS
LTHLIFGYVFNQKLTENVLPNS
LTHLTFGDDFNQIITENVLPNS
LTHLTFGWSFNQKITKNVLPNS
LTHLSFGYGFNQQITENVLPCS
LTHLTFGYEFNQELTENVLPCS
LTHLTFGYEFNQELTENVLPSG
LTHLDFGSDFNQEIT

>Crov585
MEEYIIENNILIENNILIENNILRFNYEFNEPLDKYYKILSNPK
IDTIEFGEKFNQEIDHLIPSN
IKVIKFRWWSEFNKDVNFVPES
LTEIYYGTYKNHSLEELQNLPRS
LLKLKLGDVFNHKITENVLPDG
LTHLTFGKEFNQKIVENVLPGG
LTHLTFGCEFNQKIVENVLPGG
LTHLTFVYKFNQEIVENVLPGG
LTHLTFGWYFNQQITENVLPNS
LTHLTFGNNFNQQIRENVLPNS
LTHLSFDCEFNQKLTENVLPSN
LTHLTLGRDFNQEIVENVLPNS
LTNLTFRHNFNQKITENVLPNS
LTYLEFGRNFNQQITENVLPNS
LTYLEFGRNFNQQITENVLPNS
LTYLEFGRDFNQQITENVLPNS
LTRLTFGNNFNQIITENVLPNS
LTHLTFGDDFNQIITENVLPNS
LTLLTFGKNFNQIITENVLPSS
LTHLTFGYNFNQKITENVLPCS
LTHSIFGFYFNQEIIYYNLFSYLFFNLNKTPCILPSG
LTHLTFGYNFNQKITENVLPCS
LTHLTFGNNFNQKITENVLPNN
LIWLDLGRVYNHELTQYILPNN
LKYLWLSYNYNQKIKENVLPNN
LIELSLGRVYNYELTPYILPNN
LKYLKLSDNYNQKIKENVLPNS
LIRLNLGRVYNHELTPHILPNN
LKYLRLSYNYNQK

>Crov583
MEEYIIENNILIENNILIE
NNILTFNYVFNEPLDKYYKILSNPK
IDTIEFGKKFNQEIDHLIPSN
IKVIKFRWWSEFNKDVNFVPES
LTEIYYETYKNHSLEELQNLPKS
LLKLKLGDVFNHEITGNVLPGG
LTHLTFGKEFNQKIVENVLPGG
LTHLTFGCEFNQKIVENVLPGG
LTHLTFGYKFNQEIVENVLPGG
LTHLTFGFYFNQKLTENVLPNS
LTHLTFGNNFNQQIRENVLPNS
LTHLSFDCEFNQKLTENVLPSS
LTHLIFGFYFNQKLTENILPNN
LTNLTFRHNFNQKITENILPNS
LTYLEFGRNFNQQITENVLPNS
LTYLEFGRNFNQQITENVLPNS
LTYLEFGHDFNQQITENVLPNS
LTRLTFGNNFNQIITENVLPNS
LTHLTFGDDFNQIITENVLPNS
LTLLTFGKNFNQIITENVLPSS
LTYLTFGLRFNQKITKNVLPSS
LTHLTFGYNFNQKITENVLPGG
LTHLTFRNNFNQKITENVLPSS
LTHLTFGNNFNQKITENVLPNN
LIWLDLGGVYNHELTPYILPNN
LKYLRLSYNYNQKIKENVLPNS
LIRLNLGRVYNHELTPYILPNNLKYLRLSYNYNQK

>Crov581
MEEYTIKNTILTFNDEFNEPLDKYYKVLSNPE
IDTIEFGINFNQEIDHLIPSN
VEVIGFRRNSRFNKDVNFLTES
LTEIYYGIYKNHSLEELQNLPKS
LLKLKLGNVFDHEITENVLPGG
LTHLTFGLYFNQQITENVLPGG
LTHLTFGWNFNQQITENVLPNS
LTHLTFNTCFNQKIIENVLPNS
LTHLTFGCCFNQKIVENVLPGG
LTHLTFGRDFNHKITENVLPNS
LTHLSFVFWFNQIITENVLPSG
LTHLDFGTDFNQEITENVLPNS
LTHLTFGDKFNQIITENVLPNS
LTHLTFGDNFNQIITENVLPNS
LTHLTFGDNFNQIITENVLPCS
LTHLTFGWYFNQELTENVLPNS
LKVLKIYYVNKDIILKNIDTSKIKFKIEYVK

>Crov580
MEEYIIENNILRFNDEFNEPLDKYYKILSNPE
IDTIEFGENFNQEIDHLIPSN
IKVIKFKHNSRFNKDVNFLTES
LTEIYYGKYKNHSLEELQNLPKS
LLKLKLGDVFNQEIVENVLPGG
LTHLTFGWYFNQKLTENVLPES
LTEIYYGKYKNHSLEELQNLPKS
LLKLKLGDVFNHEITGNVLPGG
LTHLTFGWYFNQKIVENVLPGG
LTHLTFGCEFNQKIVENVLPGG
LTHLTFGGEFNQKIVENVLPTS
LTHLTFGWYFNQQITENVLPNG
LTHLTFGFYFDQKIIENVLPNS
LTHLNLGYCFNQKITKNVLPNSLTHFNHY

```
>Crov577
MEEYIIENNILRFNNEFNEPLDKYYKVLSTPE
IDTIEFGDDFNQEIDHLIPSN
IKVIKFGHDSEFNKDVNFLTES
LTEIYYGIYKNHSLDEVQNLPKS
LLKLRLGHLNQELTGNVLPNS
LTHLDLGEDFNQNLTENVLPTS
LTHLTFSYCFNQKITENILPNS
LTHLTFGGRFNQKITENVLPGG
LTHLTFGWNFNQQITENVLPNS
LTHLTFGTCFNQKIIENVLPNS
LTHLTFSYCFNQKIVENVLPGG
LTHLTFGRDFNHKITENVLPNS
LTHLSFVCWFNQIITENVLPCS
LTHLTFGWNFNQQITENVLPNS
LTHLTFGDNFNQIITENVLPNS
LTHLTFGDNFNQIITENVLPNS
LTHLTFGDNFNQIITENVLPNS
LTHLTFGNNFNQKITKNVLPCS
LTHLTFGFGLSYKELTLNILPKN
LQVLQLINIDKDIILKNIDTSKIKFKIEYFK

>Crov576
MEEYIIENNILRFNDEFNEPLDKYYKILSNPK
IDTIEFGDDFNQEIDHLIPSN
IKVIKFGHDSEFNKDVNFLTES
LTEIYYGIYKNHSLEELQNLPKS
LLKLKLGDVFKHGITENVLPGG
LTHLTFGCNFNQQITENVLPDG
LTHLTFGWHFNQQITENVLPDG
LTHLTFGWHFNQQITENVLPNS
LTHLIFGYNFNQKIIENVLPNS
LTHLTFGKYFNQKITENVLPNS
LTYLTFGLRFNQKLTENVLPNS
LTHLSFGGHFNQQITENVLPNS
LTHLTFDKDFNQQITENVLPYS
LTHLTFGKDFNHKITENVLPNS
LTYLTFGTNFNQKITDNILPNS
LTHLSFGCWFNQKITENVLPNS
LTYLTFGVTFNQKITDNILPNS
LTHLTFGDRFNQKITENVLPDS
LTHLDFGTDFNQEITENVLPGG
LTHLTFGYDFNQEIVENVLPNS
LTHVFFGYMFNQKLTENVLPSS
LTHLTFKRKFKQELTLNILPDN
LKVLKIYYGNKDIILKNIDTSKIKFEIEYFN

>Crov575
MEEYIIENNILRFNNEFNEPLDKYYKVLSTPE
IDTIEFGENFNQEIDHLIPSN
IKVIKFKHNSRFNKDVNFLTES
LTEIYYGKYKNHSLEELQNLPKS
LLKLKLGDVFNQEIVENVLPNS
LTYLEFGRNFNHEITENVLPGG
LTHLTFGTCFNQKIIENVLPGG
LTHLTFEWYFNQEIVENVFPDS
LTHLKFGPNFNQKIIENVLPNS
LTHLSFYYCFNQKITENVLPNS
LTHLSFGYSFNQKITGNVLPNS
LTHLTFGGNFNQQITENVLPCS
LTHLTFGYCFYQKITENVLPNN
LKVLKIYYKNKDIILKNIDTSKIKFKIEYFK
```

>Crov574
MEEYIIENNILRFNDEFNEPLDKYYKILSNPE
IDTIEFGENFNQEIDYLIPSN
IKVIKFEPNSRFNKDVNFLTES
LTEIYYGIYKNHSLEELQNLPQS
LLKLKLGDVFDHEITENVLPGG
LTHLTFGLYFNQQITENVLPAG
LTHLTFRYKFNQEIVENVLPYS
LTHLTFGFCFNQEIKENVLPNS
LTHLTLGSYFNQEIKENVLPNS
LTHLTFGRYFNQKIKENVLPNS
LTHLSFGCWFNQIITENVLPNS
LTHLIFGFVFNQKLTENVLPNS
LTHLTFGRYFNQKIIENVLPNS
LTHLTFGNNFNQQITENVLPNS
LTHLTFGCEFNQKIVENVLPNS
LTHLTFGKKFNQIITENVLPCS
LTHLTFGWYFNQQITENVLPDN
LKVLKICCGNGDGILKNIDTSKIKFKIEYFK

>Crov573
MEEYIIENNILRFNDEFNEPLDKYYKILSNPK
IDTIEFGENFNQEIDYLIPSN
IKVIKFEPNSRFNKDVNFLTES
LTEIYYGIYKNHSLEELLNLPQS
LLKLKLGDVFDHEITENVLPGG
LTHLTFGCNFNQQITENVLPGG
LTHLTFGWNFNQQITENVLFDG
LTHLTFGWNFKQQITENVLPNS
LTHLIFGYKFNQEIIRNVLPKN
LTHLIFDYEFNQKLTENVLPNN
LTHLTFGNCFNQKITDNVLPNN
LTHLTFGSSFNQKITDNILPNS
LTHLTFGLYFYQKITKNVLPSS
LTHLSFQHKFYQKIIENVLPNS
LTHLTLGRYFNKEITENVLPNS
LTHLTFGNNFNQELKENVLPNS
LTHLTFGNNFNQIITENVLPNS
LTHLTFGDDFNQIITENVLPNS
LTHLTFGDDFNQIITENVLPDN
LKVLKIYYGTKDIILKNLDTSKIKFKIEYFK

>Crov572
MKEYIIENNILIENNILRFNYEFNEPLDKYYKILSNPE
IDTIEFGINFNQEIDHLIPSN
IKVIKFVPYSRFNKDVNFLTES
LTEIYYGKYKNHSLEELQNLPQS
LLKLKLGDVFNHKITENVLPNS
LTYLELGHNFNQKIIENVLPNR
LVHLSFGYKFNQEIVENVLPNS
LTHVFFGYMFNQKLTENVLPNS
LTLLAFGWYFNQKITENVLPCS
LTHLTVGWYFNQQITENVLPNN
LKVLKIYYGNKDIILKNIDTSKIKFKIEYFK

>Crov571
MEEYIIENNILRFNYEFNDPLDKYYKVLSNPE
IDTIEFGENFNQEIDHLTPSNIK
VIKFRWWSKFNKDVNFLTES
LTEIYYGKYKNHSLEELQNLPQS
LLKLKLGDVFNHKITENVLPGA
LTHLTFGEEFNQKIVKNVLPGG
LTHLTFGWHFNQQITENILPDG
LTHLTFGWNFNQQITENVLPDG
LTHLSFGMYFNQKITKNVLPSS
LTHVTFGLYFNQKITKNVLPSS
LTHLTFGYCFNQKITENVLPNS
LTHLTLGRYFNQEITENVLPNS
LTHLTFGNNFNQELKENVLPDN
LKVLKIYYINKDIILKNIDTSKIKFKIEYF

>Crov570
MKEYTIKNTILTFNDEFNEPLDKYYKVLSNPE
IDTIEFGENFNQEIDHLIPSN
IKVIKFVPYSRFNKDVNFLTES
LTEIYYGKYKNHSLEELQNLPQS
LLKLKLKLGVFDHEITENVLPGG
LTHLTFGVCFNQKITENVLPGG
LTHLTFGWYFNQKLTENVLPNS
LTYLTFGLRFNQKITENVLPCS
LTHLTFG

>Crov568
MEEYIIENNILIENNILRFNYEFNEPLDKYYKILSNPK
IDTIEFGENFNQEIDHLIPSN
IKTIKFNYWSQFNKDVNFLPES
IKEIYYGIYKNHSLEEVQNLPKS
LLKLKLGDVFNHEIVENVLPNS
LTHLEFGHSFNHEITENVLPGG
LTHLTFGWYFNQEIIRNVLPKS
LTHLIFGYWFNQKLTENVLPSG
LIHLTFGFYFNQKLTENVLPNS
LTHVFFGYMFNQKLTENVLPSG
LTHLTFKRKFKQELTLNILPDN
LKVLKIYYGNKDIILKNIDTSKIKFKIEYFK

>Crov567
MEEYIIENNILIENNILRFNYEFNEPLDKYYKILSNPK
IDTIEFGENFNQEIDHLIPSN
IKVIKFRWCSKFNKDVNFLTES
LTEIYYGKYKNHSLEELQNLPQS
LLKLKLGDVFNHKITENVLPNS
LTYLELGHNFNQKIIENVLPNR
LVHLSFGYKFNQEIVENVLPNS
LTHLTFSYCFNQKIVENVLPGG
LTHLTFGRDFNQKITENVLPNS
LTYLTFGLRFNQKLTENVLPNS
LTHLSFCYCFNQKITENVLPNS
LTHLSFGYSFNQKITENVLPNS
LTHLTFGYNFNQKITENVLPCS
LTHLTFGYCFNQKITENILPNN
LTYIEFGRNFNQQITENVFPNS
LTYKILMKEYIIENNILRFNDKFNEPLDKYYKILSNPK
IDTIEFGKNFNQEIDHLIPSN
IKVIKFSNNSRFNKDVNFLTES
LTEIYYGKYKNHSLEELQNLPKS
LLKLQLGDVFDHEITENVLPGD
LTHLTFGCNFNQQITQNVLPDG
LTHLTFGWNFNQQITENVLPGD
LTHLTFGCNFNQQITQNVLPDG
LTHLTFGWNFNQQITENVLPDG
LTHLSFGYKFNQEIVENVFPDS
LTHLKFGPNFNQKIIENVLPNS
LTHLTFGYCFNQKITENVLPNS
LTHLSFGYSFNQKITENVLPCS
LTHLTFGWYFNQELTENVLPDT
LKVLKIYYGNKDIILKNIDTSKIKFKIEYFNKN

>Crov566
MKEYIIENNILRFNDEFNELLDKYYKVLSNPE
IDTIEFGENFNQEIDHLIPSN
IKVIKFKYNSRFNKDVNFLPES
IKEIYHGFGKNHSLEELQNLPKS
LLKLKLGDVFDHEITENVLPGG
LTYLEFGRNFNHEITENVLPGG
LTHLTFGTCFNQKIIENVLPGG
LTHLTFGWYFNQKLTENVLPNS
LTHLSFYHCFNQKITENVLPNS
LTHFAFGWCFNQKITKNVLPNS
LIHLAFGCNFNQQITENVLPSG
LTHLDFGADFNQEITENILPDN
LKVLKIHYGNKNKDIILKNIDTSKINFKIEYF

>Crov565
MEKYTIKKTILTFNNEFNEPLDKYYKILSNPK
IDTIEFGEEFNQEIDHLIPSNIE
VIKFEPNSRFNKDVNFLTES
LTKIYYGIYKNHSLEELQNLPKS
LLKLKLGDVFNQEIVENVLPNS
LTYLELGHNFNQKIIENVLPGG
LTHLTFGYEFNQKIVENVLPGG
LTHLTFGIEFNQEIVENVLPVG
LTHLTFGWYFNQKLTENVLPNS
LTHLTFGTCFNQKLTENVLPNS
LTHLIFGFVFNQKLTENVLPNS
LTHLSFGYIFNQKITENVLPNS
LTHLSFGCGFNQQITKNVLPDS
LTHLSFGYGFNQQITENVLPSG
LTHLDFGSDFNQEITENILPNS
LTHLTFGYKFNQELTENVLPDN
LKVLKIHYGNKNKDIILKNIDTSKIKFKIEYFK

>Crov564
MEEYIIENNILRFNDEFNEPLDKYYKILSNPK
IDTIEFGENFNQEIDYLIPSN
IKVIKFEPNSRFNKDVNFLTES
LTEIYYGIYKNHSLEELQNLPKS
LLKLKLGDVFNQEIVENVLPNS
LTYLELGHNFNQKIIENVLPGG
LTHLTFGCEFNQKIVENVLPGG
LTHLTFGIKFNQEIVENVLPNS
LTHLSFGYIFNQKITENVLPNS
LTHLSFGYGFNQQITKNVLPGG
LTHLTFGWYFNQKLTENVLPKS
LTHLIFGYVFNQKLTENVLPNS
LTHLTFGDDFNQIITENVLPNS
LTHLAFGWCFNQKITKNVLPNS
LTHLSFGYSNQQITKNVLPNS
LTHLSFGYGFNQQITKNVLPNS
LTHLSFGYGFNQQITENVLPSG
LTHLDFGSDFNQEITVNILPNS
LTHLTFGTSFNQKITENVLPNS
LTYLTFGLRFNQKITENVLPCS
LTHLTFGWYFNQELTENVLPDN
LKVLKIYYGNKDIILKNIDTSKIKFKIEYF

>Crov563
MEKYTIKETILRFNYEFNEPLDKYYKILSNPK
IDTIEFGETFNKEIDHLIPSN
IKVIKFGWYTDIKIVDFRFYAAQFNKNVNFLPES
IKEIYYGHNKNHSLEELQNLPKS
LLKLQVGSKFNQQITENVLPNS
LTHLAFGRRFNQKITKNVLPNS
LTHLSFGYGFNQQITENVLPSG
LTHLDFGSDFNQEITENILPNS
LTHLTFGYEFNQELTENVLPDN
LKVLKIYYINKDIILKNIDTSKIKFKIEYF

>Crov562
MKEYTIKNTILTFNDEFNEPLDKYYKVLSNPK
IDTIEFGRNFNQEIDHLIPSN
IKVIKFELWSKFNKDINFLTES
LTEIYYGPNKNHSLEELQNLPKS
LLKLKLGDVFDHEITENVLPGG
LTHLTFGECFNQKITENVLPNS
LTHLTFGWHFNQQITENVLPNS
LTYLSFGCRFNQKITENVLPNS
LTHLTFGTCFNQKIIENVLPNS
LTHLTFGWNFNQQITENVLPNS
LTHLSFGCWFNQKITENVLPNS
LTHLAFGWCFNQKITKNVLPNS
LTHLTFGNDFSQKITENVLPDN
LKVLKIYYGNKDIILKNIDTSKIKFKIEYFK

>Crov561
MEEYIIENNILRFNDEFNEPLDKYYKVLSNPE
IDTIEFGINFNQEIDHLIPSN
IKVIKFGGNSEFNKDVNFLTES
LTEIYYGKYKNHSLEELQNLPQS
LLKLKLGDVFNHKITENVLPNS
LTYLELGHNFNQKIIENVLPNR
LVHLSFGYKFNQEIVENVLPNS
LTHLTFSYCFNQKIVENVLPGG
LTHLTFGRDFNQKITENVLPCS
LTHLTFGWYFNQQITENVLPNS
LTHLSFGCWFNQKITENVLPNS
LTHLAFGWCFNQKITKNVLPNS
LTHLTFGNDFNQQITENVLPNS
LTHLTFGYNFNQKIIENVLPNS
LTHLIFGFLFNQKLTENVLPNS
LTHLTFSYCFNQKIVENVLPGG
LTHLTFGYEFNQELTENVLPSG
LTHLDFGADFNQEITENILPDT
LKVLKIYYKNKDIILKNIDTSKIKFKIEYLFYISYQF

>Crov559
MEEYIIENNILTFNYEFNEPLDKYYKILSYPE
IDTIEFGYKFNQEIDHLIPSN
VKVIKFGQYSKFNKNVNFLPES
IKEIYYGPNKNHSLEELQNLPKS
LLKLKLGDVFDHEITENVLPSG
LTHLTFGYEFNQEIVENVLPGG
LTHLTFGEEFNQKIVENVLPGG
LTHLTFGWYFNQKLTENVLPNS
LTHLTFGTCFNQKLTENVLPNS
LTHLTFGTCFNQKITENVLPNS
LTHLAFGWCFNQKITKNVLPNS
LTHLTFGNDFNQKITENVLPNS
LTYLEFGRNFNQEIVENVLPKS
LTHLIFGYVFNQKLTENVLPNS
LTHLTFGDDFNQIITENVLPNS
LTHLTFGYGFNQQITENVLPCS
LTHLTFGYEFNQELTENVLPSG
LTHLDFGSDFNQEIT

>Crov558
MEEYIIENNILRFNDEFNEPLDKYYKVLSYPEI
DTIKFGRNFNQEIDHLIPSN
IKVIKFEPNSRFNKNVNFLTESLT

>Crov029
MNEYTIKKSILTFNNTFNKQLNKYYKVLSNPK
IDTIKFGEYFNQEIDHLIPSN
IKVIIFSYKSKFNKDVNFLPET
IEEIYYGDNKNHHIGELLNLSKS
IVKLQLGGKFNQELKENVLPKS
LKLLIFGKEFNQELKENVLPKS
LKLLIFGKEFNQELKENVLPKS
LKLLIFGKEFNQELKENVLPKS
LKLLIFGKEFNQELKENVLSNN
LTYLTFGKKFNHEIKENVLPKS
LKFLTFGKEFNQELKENVLSNN
LTYLTFGKKFNHEIKENVLPKS
LKFLTFGKEFNHEIKENVLPKS
LTYLKFGSNFNQELKENVFPNG
LTLIRSSTNQKITKNVLPSLPSLPSLPSLPSLPSLPSLPSLPSLPSLPTSLAN
LTNLTFNNMLNQGYVLPKSLQLFKI

>Crov028
MEEYIINNTILTFNDTFNKPLDKYYKMLSNPE
IYTIEFGENFNQEIDHLIPSN
IKVIKFGWWSKFNKDVNFLTESIE

>Crov027
MEEYIIENNILRFNDEFNEPLDKYYKVLSNPE
IDTIEFGRNFNQEIDHLIPSN
IKVIKFEPHSRFNKDVNFLPES
IKEIYYNSNKNHSLEELQNLPKY
LLKLGLGGNFEELTENVLPNS
LTHLSLGTYFNQKITVNVLPNS
LTHLTFGWYFNQELKENILPNS
LTHLIFGKNLTFGGRFNQELKEKVLPKN
LTHLTFGGRFNQELKENVLPNS
LTHLEFGNNFNQKISENVLPNC
LTHLVFGFYFNQEITENVLPNS
LTHLTFDGHFNQKLTENVLPNS
LTHLTIRWYFKEIKENILPDN
LKVLKIYYQNKDEILKNIDTSKIKFKIEYLF

>Crov002
MNIIMSKLEIYTVDDLSKITPD
ITQLYFTTNFNSPIDNLPYW
IKNIKFEWNSKFDCQVDMLPEG
LKELILGKHKNPLPDELRNLPKS
LTHLTFGCDYNQKIEENVLPES
LTHLTFGSDYNQKIDANVLPKN
LTHLTFGIHYNQKIEENVLPES
LTHLTFGYSYNQKLDENVSPKN
LTHLTFGFCYNQKIEENVLPES
LTHLTFGYSYNQKLDENVSPKN
LTHLTFGYCYNQKIEENVLPKS
LTNLTFGYCYNQKIEENVLPES
LPHLTFGWGFNQKIEENVLPKN
LTHLTFGTYYNQKIAENVLPKN
LTHLEFGWHYNQKLDENVLPKS
LTHLTFGTNFNQKIAENVLPKS
LTHLTFGIHYNQKIEENVLPES
LTYLIFGYCYNQKIEENILPES
LTYLTFGENHNQKIEENVLPES
LTYLTFGYCYNQKIEENVLPKS
LTYLTFGYCYNQKIEENVLPKS
LTYLTFGYCYNQKIEENVLPKS
LTYLTFGENYNQKIEENVLPES
LTHLTFGYCYNQKIEENILPES
LTYLTFGENYNQKIEENVLPES
LTHLTFGTEFNQKIEENVLPQS
LKEITISIYYNYNLPDDIIISRI

>Crov001
MSKLEITISDDLNKITPNITKLDFTSEFDSPTDWECVRLIDSPIDSPIAGPSACPTDR
ECVSPIGSPIVRDLANRDIVLPKS
LTHLTFGTNFNQKIEENVLPKS
LTHLTFGGDYNQKIEENVLPKS
LTHLTFGFYYNQKIEKNVLP

>Crov539
MTLVIKTNEDLNKLNDN
IHTLTIGANFNQPIEHIKWPKL
LTTLTFEWYFDQPIENVKLPDS
LTTLTFGYSFNQPIEKVKWPKT
LAFLTFGYKFNKPIEKVKWPDS
LTTLIFEENSLFDQSIEKIKWSNS
LTTLIFGWNFNQPIENVEWPES
LTTLVFNEDSIFNQPIENVKWPKL
LKTIIFGCHFNHPIENVKWPGS
LTTLIFGDDFNQPFENVILPKS
LTNLTFGPNFNQPLNFLPES
LKNITITTNYQQNLYNLPSS
LNCIKIISYKRTYEHIVNVLPEHLKKKVIKI

>Crov631
MDIIYTSGLYKHLVDNTNSIIINKNNINVLEDDSSML
LTTVIFNDNFNEKIDNVKFPDS
ITSIIFGLEFNQSLDNVRWPK
LLKYLHFSYYFEESFDFIPES
VECLSIEENHVLSFLNLPFN
LKEIKINFAHNKIKKLDEYNTNYDLYKKALPSY
LSNIKIILNNEIYTHFVGDYTHTFDDTTSIEINKNNINVLYNKSSMS
LTTLIFSDTFNEQIETFKFPDS
ITSIIFGWNFNQSLENVSWPKS
LKYVHFCYYFDWSLDFLPTS
LEYLSIEYNYGLDSMYFKNLPKN
LKKLRINFQRDRITSWDEHDFYYDRYKEALPAHLSNVEIIIN

>Crov541
MSLVIQTSEDLNKINNS
IHTLTFGCSFNQPIENVVWSES
LTTLTFGSYFDQPIENVTWPNS
LTTLTLGHNFNQPIKNVVWPES
LTTLTFNVFFNQPLEKVTWPES
LTNLTFGYYFNQPIEKVKWPKS
LTTLSFGYNFNQPIEKVRWPES
LTTLTFCEYFNQPIENVIWPDS
LTTLTFGFEFNQQLDFLPES
LKELTLTSNYKQNLYNLPSS
LNSIKIITFQKPIVHILNVLPEHLKNQVILS

>Crov531
MSLVIHTSEDLNKINNS
IHTLTFGERFNQPIENVVWPES
LTTLTFGSYFDQPIENVTWPNS
LTTLTLGHNFNQPIKNVV

>Crov527
MSLVINTLKDLIKINDNYN
LHTVTFGYEFNQPINKVKLSNS
VTTITFGSRFNQPIENVTWPKL
LTTLTFGVEFNQPIENVILPNS
LTTLTFGSRFDQPIENVKWPKS
LTTLSFGWDFNQSIENVSWPHT
LSTLSLKGKFNQSIKSTCWYTFILGYSTHSEKMILPIS
LTTLTFSSYFNKSIENVVWPES
LTTLTFGNKFNQPIENVTWPES
LTTLTFGNKFNQPIENVTWPES
LNTLTFEQDFNQPIENVTWPES
LNTLTFGKNFNQPIEKIKWSQS
LTTLTFDEYFDQPIENIKWPES
LTTLIFKGVNQPIENVIWPKS
LTTLAFGDTFNQSINKVKWPES
LTTLIFGQLFNQPIENVKWPKS
LTTLTFGEKFNQPIENVTWPKS
LTTLTFGEKFNQPIENVKWPKS
LTTLTFGEKFNQPIENVKWPKS
LTTLTFGKKFNQPIEKVKWPKS
LTSLTFGWDFNQQIENVKWPKS
LTSLTFGWDFNQQIEKVKWPES
LTTLTFGCEFDQLIENTKLPDS
LTNLNFSVLFNQSLKKVKWPSS
LTTLTFGHNFNQPIDNIKWPKS
LTTLTFGEKFNQPVDNVIWPKS
LTTLKFGDEFNQPLDFLPES
LKNIEITTFYEKNLYNLPSS
LDCINVPIDYDSDADDDDFIYKFSEHLQNKVNFI

>Crov514
MSIIIHNNNVLSHINSNIHTITCDFDQPIENLEIPKT
ITTIIFCNSFNQPIENVKWPES
LTTLTFGRKFDQPIEKVQWPKS
LTTLTFGREFDQPIEKVQWPES
LTHLSFGDSFNQPIENVIWPKS
MTNLTFGYNFDQPIENVKWPEL
LTTLKLGYTFNQPIDYVKWPNL
LSTLIFGGFFARSIKKVKWPES
LTTLTFGWRFNQPIENVKWPKS
LAYLTFDKHFNQPIEKVIWPES
LTTLTFGYDFNQSIEKVIWPES
LTTLTFGYTFNQPLDYVKWPKS
LTTLIFGVKFNQLLDFLPET
IKKLTIASKYNQNLDNLPLHLTKNKIKHNNYIVFG

```
>Crov513
MTFHGRFNQPINNVKWPKS
LTALSFGNNFNQPLDFLPES
LKELTLNSTYNHDLSNLPSG
INRIQLNYHNSYRHKVPTHLKNKLYPINELYKK

>Crov507
MQQKNIKEYIQVPSFNKNS
LYNLYFGRNDSVDLSNIPNY
ITHIYIDYDNFFILSSTSCPSLTHVRWPSH
LESIEFPIHFNQPIEHIIFPDS
LKRMVLTQGDSFFNYPIYRFKWPKS
LIELTFYQRNHSLKGIQLPNLKYLNICGEYNLKLPDS
IETLYLQRFVPLPSHEVSKELLLPKS
LTKLVFGSGFDDSLEIIKKWPDS
LTTLTFGYNFNQAIEKVKWPKS
LTTLTFGYNFNQAIEKVKWPES
LTTLTFGYNFNQAIEKVKWPKS
LTTLTFGYNFNQAIEKVKWPKS
LTTLTFGYNFNQAIEKVKWPES
LTTLTFDDTFNQPIENVCWSKS
LTTLTFGENFKQTIEFLPEN
LEKLKLKLKLKIVDMSYMYVTPNLPNS
LKLIEVYMADYIQPFINNMTNILKEHFTVETHPFIKNWFYFIRKYQ

>Crov427
MTFLLTPTYNCTDIISEK
IDTIIIKDGTQSIIEKIKWPKT
VTTLILDNLFNESLDNISWPES
LTTLNLSYNFNQPIENIKWPVS
LTVLNFGYDFNQPIENIIWPES
LKSISFLYDCQFNHPIEHIQLPNS
LTTLTFNKNFNQPIEKVKWPKS
LTTLSFGYEFNQPIEKVKWPKS
LTTLIFNGCFNKPIEKVKWPNS
LTTIIFKGKFNQPIKKVKWPSS
LTELTFNDNFNQSINKANLPNS
LKKLTLNSNFNYPLDNIKWPSS
ITILTWGKWSQFNQSLDNMRWPES
LTTLTLGDKFDQSIEKVKWPAS
LTSITFYWSSLFNQPIEKVKWPES
LTTLCFNDIFNQPIEKIKWPKS
LSNITFGDAFNQPINNVLWPNS
LTSLNLPYTKHITN
LTSLVTLKNLNIFFHDNYTCSIAQIKWPPN
ITSLIFDGNFDQSIDTITFPSS
LKKLTICYHFDQSIDNVLWPKS
LTTLNLSHMFNQPIDKVKWPDS
LKTISFGESFNQPIENVTWPKS
LLTLSFGDNFNQPIEKAQLPDS
LATLIFYSDFNQPIENVKWPTS
LTTLNLGCKFRQSLDFLPQS
LKEIFIYDMHIHDLSNLPSS
INKINIYKYKNYNYIYIINKLPDILKSKVVLLKVE
```

>Crov409
MKLIINSEYDYSSNIND
VCDIIIQELNCPIENIKWPKSS
ISLTFEYFFNQPIEKVKWPDS
LTSIIFKSDAFFNQPIEKVKWPNS
ITTLIFDGCYFNQPIEKVKWPSS
LTTLKFGSKFDQPIEKVKWPSS
LTTLIFESNLKQPIENIKLPNT
LTALTISAYFNPSCENIKFPES
ITTLNLKWFDTPLENIIWPKS
LTSLTFGDNFNQPIENVTWPNS
LTTLSFGRYFNQPIEKVTWPKS
LTTLTFGWFFNQPIDKVQWPKF
LTSLHFSGSYTSFNQPIENVQWPDS
LMTLDFGEEFNQSIEKVKWPES
LTIITFGDKFNQPIEKVKWPES
LTTLTFGRHFNQPNVKCPDS
LTTLSFGRHFNQPIDNVKWPDS
LTTLSFGRHFNQPIEKVKWSKS
LTTLTFDGDFNQPIENVKWPES
LTTLEFGCDFNQPIEKVILPES
LTTLIFGDDFNQPIEKVKWPKS
LNILTFSENFNQPIEKVILPES
LTTLIFGHNFNQPIEKVKWPCS
LIKLHFDWEFDQPIEKVKWSNS
LTTLSFVGNFNQPVDYIKWPNS
LTYLHFNDGLCNEDEIFKGISIFNQPLEFLPEN
LKKLTLTCHYKHNLFNLPSSIQEINVIMNYFNEMSYYDEEADLDDEDDNDIIYNIRVENFKKRVADNHLYKLNFII
LAT

>Crov408
MARS
LTTLNLGCNFDQPLDKVKWPAS
LTTLNLGCNFDQPIENVQWPDS
LITLNFSNTTYEAVFNQPIENVKWPKS
LTTLYFDDIFDRPIENIKWPHS
LTNLTFGRFFNQSIENITGPESLPNLAFGHNFN

>Crov406
MDSLITNLVCRQVLYNIYHFTKKIKLHYLIKTITFDRHMTMLIKNINFSKN
TTSLIFKENYPIPLENIEWPSS
ITSLSFKYNFNQSLNFIMWPKL
LTTLNIELDNNQPIDMINFPES
LTSLTLGNKFNKPIDKVQWPQS
LTSLTLGGNFNKRIDKVKWPQS
LTSLSIGNLFNQSLKNVQWPKS
LTNLSLGENVYVNPKTKIDIPSS
INTLKLSVDYSIFNNIKFPSK
LTTLHIKLINWNNITLLNLLLPSTN
INTDVLLDYIIFPSSLNTLIFECDETIINPLDNVIWPES
LTNLTFGLTFNQPIENIKWPKS
LTALTFGMMFNQSIEKVIWPKS
LTALTFGMMFNQSIEKVIWPKS
LTALTFGMMFNQSIEKVIWPKS
LTALTFGDDFNQSIKNIKWPKS
LTALTFGMMFNQSIEKVIWPKS
LTALTFGSCFNQPIEKVIWPNS
LTTLTFGYNFNQSIENVYWPES
LTNLTFGTRFNQPIENIRWPNS
LTTLAFGVKFTQLLDFIPEN
LKKLIIYYKYPNSLFNIPIN
LNNLEILIGNNEPKNCIELNNILNDFTDELPFYLKNKLSYKLLDFKNSIFL

>Crov124
MVLRINSTFNYLKKISNN
ITTIIIEDNLNFPINKIKWPNS
LVTVIFSEYFNQSLDGIIWPNT
VQYFHFGQYFNQPLDKIIWPES
FTTLSFRQNFNQPLDNVIFPPT
LKTLIFGMYFNQPIENVKWPDS
LSILKFNESRFNYPIEKTKWPKS
LTTLTFGYYFNQPIEKIKLPDS
LQTFIIGGKFNYPVENVKWPKS
LTNLSLIGKFNYPLDFLPDS
LKKLSLSIRYTHNLENLPDN
IEYIEVHILIDVDEYYDFSIKLSQKLKNKVIYIYD

>Crov123
MNILDDNNNFIYNGKLVQLPKL
LTNLTINDKNQLIIKNIILPELV
VTQIKNYNNNNNLLTNVEWPKS
LTSLTFGWNFNQPIDKVAWSNS
LLTLIFGNHFNKSIENVIWPES
LTSLTFGYNFDQPMKMLYGLNL

>Crov114
MSLKISDITDLQNINDK
IHTIIFDDDFDQPIDNVKWPDS
LTTLTFGFHFNQPIEEVKWPDS
LTTLTLEYKFNQPIEKIKWPNS
LTTLTFGWDFNQPIEKVKWPDS
LTTLTFDRDFNQPIEKVKWPVS
LTTLSIVSIFNQPINKIKWPNS
LTSINLIASFNQPIEDVKWPDS
LTTLTFGAWFNQSIEKIKWPNS
LTTLSFGHNFNQPLDKIKWPNS
LTTLSFGHNFNQPLDKIKWPNS
LTTLTLGEKFNQPIDNIKWPDS
LTTLTIGWIYNKLLDKIKWPEL
LTTLTLGYKFNQPIDNIKWPDS
LTTLTFGNDFNQPIEKVKWPNS
LTTLTFQHYLNQPIEKVKWPDS
LTTIIFFGNFNQPIKKVKWPDS
LTTLTLGWNFNQPIENVKWSSS
LTALTFTRDFNQPIENVKWPNS
LTTLTFGENFNQPIETVKWPDS
LTTLTFGENFNQPIETVKWPDS
LTTIIFRGYFNQPIEKVKWPDS
LTTLTFNNFNQPIENVKWPNS
LTTLTFGYNFNQPIEKVKWPDS
LTTLTFRNNFNQSIEYIKWPVS
LTTLTFGEGFNQFLDFLPET
LTKLNLSKTYKLKLDNLPIKTNLNYKDF

>Crov068
MYAPQLNCTNNCDIDNN
IQNIIIEKNLNIPIENINWPKS
LINLSLIKKFNQPINNIRLPEL
LNTLTFGEYFNQPIDNIIWPES
LSKITFGKNFNQPIENVIWPDS
LTSITFGRCFNQPIENVIWPDS
LITLKFNYYSQFNQPIEKINWPKY
FTTLIFEEDSLFDQPIEKINWPKS
FTTLIFEEDSLFNQPIEKIKWPES
LTNLTLRDTFDQPIEKVKWPKS
LTTLDLGCNFNQPIEKVKWPES
LTTLALGYKFNQPIEKVLFPKS
LTTLYFSDKFNQIINNIVWPEL
LDELTFGVNFNQSINKVKWPES
LSTLKFGNNFNQPINKVQWSNS
LTTLIFSGWFNQPIENVAWPKS
LTTLIFSGRFNQPIENVIWPKF
LETLTFGNEFNQPIKNVKWSNS
LTTLNFSREFNQPIEKIKLPSS
LTELYFGKHFNQPIKNVKWPES
LTTLTFGDDFNQPIKNVKWPES
LTTLTFGNNFNQPIENIKLPKS
LTVLSFGWDFNQSIEKVIWPES
LTTLTFDKEFNQSIEKVIWPES
LTTLTFNGRSEFNQPIENVKWPIS
LTRLIFGIKFNQSLDFLPES
LEEITLPQTYKYDLYNLPTS
LKYIYIKNDNYNHGNCTIVNKLPLHLKNKVKYIS

>Crov547
MSKLEITISDDLNKITPN
ITKLDFTSEFDSPTDWECVRLIDSPIDSPIAGPSACPTDRECVRPIGSPIDSSIAGPSAR
PLDWEFDRPFAGPISGPSAR
PLDVEFDRPFAGPISGPSAR
PLDVEFDCPNASPISNSSAR
PLDLEFDCPNASPISNSSAIPHD
LKFDSPLAGPISSPIASP
ISSPIASPIVRDWDNIEIVLPQS
LTRLTFGYNFNQKIEENVLPKS
LTILKKYNHGLLIDKNLF

>Crov545
MSIIIKTLEDLEKITND
IHILTFGKDFNEPIEKIKLPKS
LRKIVFGYCFNQPIEKVKWPNT
LTILSFGYEFNQPIEKIKLSKS
LRKIVFGYCFNQPIEKVKWPNT
LTSLTFGYHFNQPIKKIKLPKS
LRKIVFGDYFKQPIEKVKWPNT
LTILSFGYEFNQPIEKIKLPKS
LRKIVFGYCFNQPIEKVSWPGL
LTKLTFGQDFDQPIENVNWPES
LTKLPFGQDFNQPIEKVKWPKS
LTTLSFGYNFNQPIEKVRWPES
LTTLTFCEYFNQPIENVIWPDS
LTTLTFGFEFNQQLDFLPES
LKELTLTSNYKQNLYNLPSS
LNSIKIITFQKPIVHILNVLPEHLKNQVILS

```
>Crov542
MDIIYTSGLYKHLVDNTNSIIINKNNINVLEDDSSML
LTTVIFNDNFNEKIDNVKFPDS
ITSIIFGLEFNQSLDNVRWPKL
LKYLHFSYYFEESFDFIPES
VECLSIEENHVLSFLNLPFN
LKEIKINFAHNKIKKLDEYNTNYDLYKKALPSY
LSNIKIILNNEIYTHFVGDYTHTFDDTTSIEINKNNINVLYNKSSMS
LTTLIFSDTFNEQIETFKFPDS
ITSIIFGWNFNQSLENVSWPKS
LKYVHFCYYFDWSLDFLPTS
LEYLSIEYNYGLDSMYFKNLPKN
LKKLRINFQRDRITSWDEHDFYYDRYKEALPAHLSNVEIIIN
```

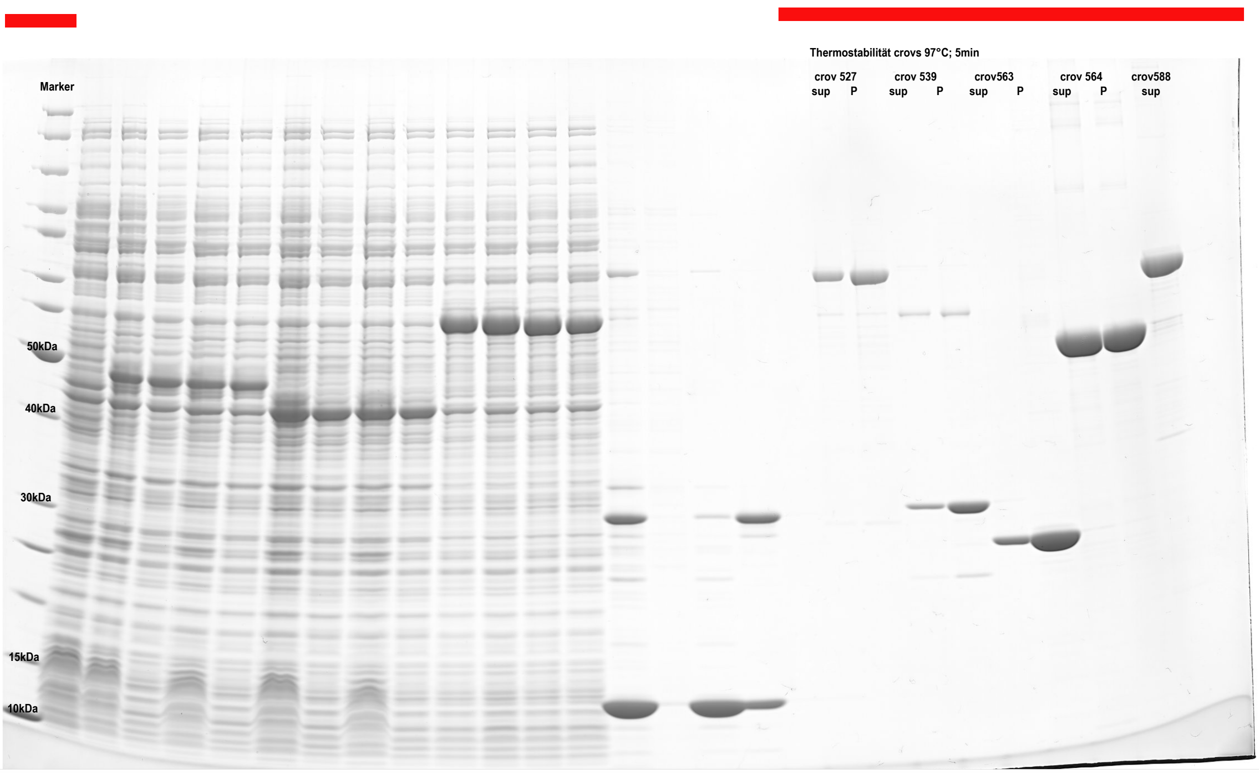

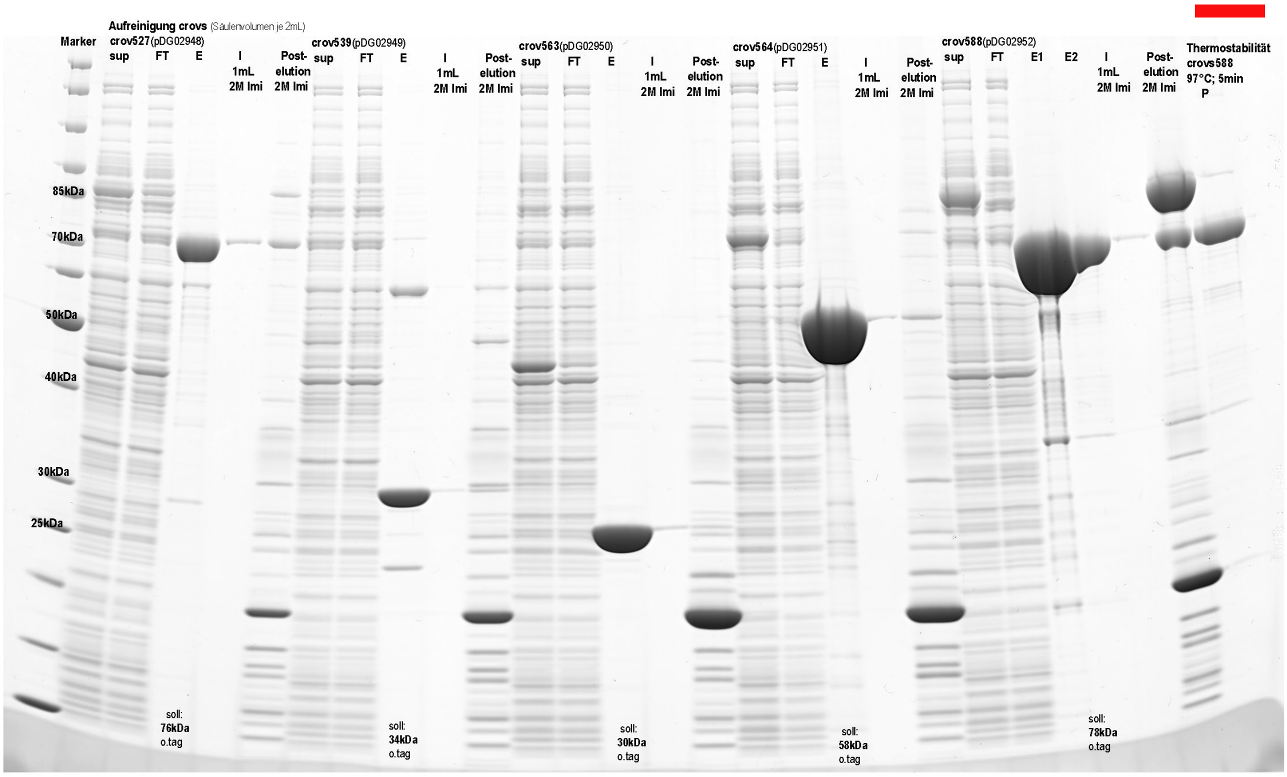

Figure 1(a) was cropped from the following 2 gels
